# Cortical bone adaptation and mineral mobilization in the subterranean mammal *Bathyergus suillus* (Rodentia: Bathyergidae): effects of age and sex

**DOI:** 10.7717/peerj.4944

**Published:** 2018-06-11

**Authors:** Germán Montoya-Sanhueza, Anusuya Chinsamy

**Affiliations:** Department of Biological Sciences, University of Cape Town, Cape Town, Western Cape, South Africa

**Keywords:** Intracortical porosity, Skeletal homeostasis, Histomorphometry, Sexual dimorphism, Cortical bone, Resorption cavities, Fossorial adaptations, Bone microstructure, Bone compactness

## Abstract

The patterns of bone modeling and mineral mobilization (skeletal homeostasis) among mammals other than humans and laboratory rodents are still poorly known. In this study we assessed the pattern of bone formation and bone resorption in the femur of a wild population of Cape dune molerats, *Bathyergus suillus* (*n* = 41) (Bathyergidae), a solitary subterranean mammal with a marked extended longevity among rodents, and which also lives in a naturally deficient state of vitamin D. In order to determine ontogenetic and sex effects on histomorphometric parameters of transversal undecalcified bone sections, two-way ANOVA, linear mixed-effects model and regression statistical analyses were performed. During ontogeny, *B. suillus* increased their cross sectional area, cortical area and cortical thickness, and most importantly, they showed scarce endosteal bone resorption which resulted in a retained medullary cavity size during ontogeny. This resulted in a positively imbalanced bone modeling, where bone formation considerably surpasses bone loss by almost 100-fold in adulthood. This differs markedly from other terrestrial mammals with relatively thin cortical walls. Regarding bone loss and remodeling, three main processes involving intracortical resorption were observed: modeling-related bone loss in early postnatal growth; secondary osteon formation occurring in both sexes; and subendosteal secondary reconstruction observed only in females. The latter is accompanied by females having six-fold more relative bone loss than males, which is evidenced by the development of enlarged resorption cavities (RCs) distributed circumferentially around the medullary cavity. Males have smaller, more circular and randomly distributed RCs. In general, our data indicate no age-related decline in mineral content in *B. suillus*, and provides strong support for a pattern of sexual dimorphism in skeletal homeostasis, similar to that occurring in humans and other mammals, with females losing more bone throughout aging as compared to males due to reproductive factors. Interestingly as well, despite the high mechanical loads experienced during burrow construction, bone remodeling in *B. suillus* is kept at very low levels throughout their lifespan, and dense Haversian tissue never forms. This study represents the first comprehensive assessment of skeletal homeostasis in a subterranean mammal, and it enables a better understanding of the complex processes governing the acquisition and maintenance of bone properties in this species with extraordinary fossorial adaptations.

## Introduction

An important goal in bone research is to understand the interrelationship between mineral homeostasis and bone modeling dynamics (i.e., uncoupled bone formation and resorption). The interaction between these processes and specific adaptations (e.g., reproductive and/or biomechanical) are also relevant to understand how bone structure is maintained with aging (Yingling & Taylor, 2008; Lanyon, Sugiyama & Price, 2009; Macica et al., 2016). However, the patterns of mineral mobilization (skeletal homeostasis) and bone modeling in mammals have largely focused on humans, non-human primates and rodents (Duque & Watanabe, 2011; Allen & Burr, 2014). Similarly, the quantification of mineral dynamics in mammals has been mostly carried out on domestic and captive specimens under laboratory conditions and generally using biological markers of bone turnover (Starič, Nemec & Zadnik, 2012; Allen & Burr, 2014; Garnero, 2014). Thus, little is known from other mammals, especially those from feral populations. The methodologies used in these analyses are focused to assess systemic mineral homeostasis but rarely specify which bones are more prone to undergo catabolic activity (Allen & Burr, 2014), thus making it difficult to identify which skeletal elements may be compromised when faced with particular life history conditions (e.g., reproductive events and/or constant mechanical loads). The rapid expansion of the medullary cavity typically observed during early ontogeny of surface-dwelling (terrestrial) mammals also restricts the analysis of skeletal homeostatic dynamics, since most of the early deposited bone tissues are resorbed (Castanet, 2006).

African molerats (AMs) (Bathyergidae and Heterocephalidae) are a large and speciose group of subterranean rodents that may help to understand some of these processes. They construct extensive burrows that are used for foraging and reproduction, whereby they are capable of enduring hypercapnic and hypoxic conditions, and hence rarely exposed to sunlight (Bennett & Faulkes, 2000; Jarvis, 2003). As a result of this extraordinary lifestyle, AMs show remarkable adaptations such as their extended longevity, which represents the highest “maximum species lifespan” among rodents (Dammann & Burda, 2007) and a mineral metabolism which seems to be unique among mammals (Buffenstein, 2000). Calcium is obtained via passive non-saturable intestinal absorption and apparently independent of vitamin D, as demonstrated by their low levels of 1,25-dihydroxyvitamin D_3_ (calcitriol) (Pitcher et al., 1992; Buffenstein et al., 1994; Buffenstein & Pitcher, 1996). This pleiotropic molecule, which is synthesized in the skin of many (terrestrial) mammals via ultraviolet radiation, plays varied roles in bone metabolism (Buffenstein, 2000; Feldman et al., 2010; DiMeglio & Imel, 2014). Calcitriol improves the efficiency of intestinal calcium absorption, contributes to the maintenance of bone mineral, increases calcium mobilization from bone (resorption) and also maintains adequate calcium and phosphate concentrations to promote normal bone mineralization (Feldman et al., 2010; DiMeglio & Imel, 2014; Anderson, 2017). Deficiencies of this metabolite cause rickets in children and osteomalacia in adults, resulting in reduced matrix mineralization of bones and increasing their susceptibility to low energy fractures (Eriksen & Glerup, 2002; Anderson, Turner & Morris, 2012; DiMeglio & Imel, 2014).

Calcium renal reabsorption in AMs is also highly efficient (Pitcher & Buffenstein, 1994, 1995; Buffenstein, 2000) and results in a positive systemic mineral flux where calcium intake is greater than its loss (Skinner, Moodley & Buffenstein, 1991; Pitcher et al., 1992; Buffenstein, 2000). As a result, AMs have a tight regulation of serum Ca^2+^ concentration (Buffenstein, 2000), with excess of calcium hypothesized to be stored in their teeth and bones (Skinner, Moodley & Buffenstein, 1991; Buffenstein et al., 1995; Buffenstein & Pitcher, 1996). Mineral content obtained from ash weight and atomic absorption spectrophotometry from bone and teeth of *Fukomys (Cryptomys) damarensis* has increased when calcium is supplemented (Pitcher, Sergeev & Buffenstein, 1994). However, when vitamin D or controlled exposure to sunlight is administrated to this species, no changes are observed in bone mineral density or calcium balance (Pitcher, Pettifor & Buffenstein, 1994). These studies indicate that mineral homeostasis in AMs is maintained by regulating bone mineral deposition and that they may have low requirements for 1,25(OH)_2_D or have evolved vitamin D independent bone metabolism (Pitcher, Pettifor & Buffenstein, 1994; Pitcher, Sergeev & Buffenstein, 1994; Buffenstein, 2000; Pinto et al., 2010; Edrey et al., 2011).

Until now, there has hardly been any histological and histomorphometric studies on the skeleton of AMs. Nonetheless, qualitative descriptions have found that the cortical walls of long bones of some AMs, and other fossorial species, are much thicker than those of terrestrial mammals of similar size, which is proposed to be an adaptation to withstand biomechanical stresses during burrow construction (Chinsamy & Hurum, 2006; Pinto et al., 2010; Montoya-Sanhueza, 2014; Montoya-Sanhueza & Chinsamy, 2017). These earlier anatomical studies support the mineral reservoir hypothesis in AMs and also demonstrate that they do not exhibit any pathologies usually associated with low calcitriol concentrations in terrestrial mammals, but rather that they have highly efficient mineral homeostasis with positively balanced bone gain (Buffenstein, 2008; Pinto et al., 2010; Montoya-Sanhueza & Chinsamy, 2017). In this sense, Pinto et al. (2010) have suggested that the eusocial naked molerat (NM) *Heterocephalus glaber* has sustained bone quality throughout most of its ontogeny.

It appears that the lack of extensive endosteal bone resorption is one of the main causes generating thick cortical walls in AMs (Montoya-Sanhueza & Chinsamy, 2016, 2017), although no quantification of these catabolic processes have yet been assessed. Thus, the magnitude and extent of bone resorption in AMs during ontogeny are also unknown. Adult individuals of Cape dune molerats (CDMs) *Bathyergus suillus*, have showed scarce endosteal resorption and some degree of intracortical resorption, with sex differences in the later (i.e., accentuated in females) (Montoya-Sanhueza, 2014; Montoya-Sanhueza & Chinsamy, 2017). Similarly, Pinto et al. (2010) briefly described that non-reproductive NMs (subordinates) lacked endosteal or intracortical resorption, whilst advanced stages of lactating females showed resorption cavities.

In general, these data suggest some similarities as well as some differences in the homeostatic dynamics of the skeletal system of AMs when compared to other terrestrial mammals. In terrestrial mammals, mineral mobilization becomes more pronounced with ageing (osteopenia), especially at endosteal surfaces, which generates bone cross sectional profiles with relatively large medullary cavities and thin cortical walls (Carrier, 1983; Heinrich, Ruff & Adamczewski, 1999; Lammers & German, 2002; Castanet, 2006; Young, Fernández & Fleagle, 2010; Silva & Jepsen, 2013; Bala, Zebaze & Seeman, 2015; Pazzaglia et al., 2015).

In several ways, the present study fills the gaps identified above by assessing femoral cortical growth and skeletal homeostasis of a wild population of *B. suillus*, a sexually dimorphic, solitary seasonal breeder and the largest extant subterranean mammal (>2 kg) endemic to the Western Cape of South Africa (Jarvis & Bennett, 1991; Hart et al., 2006, 2007; Bray et al., 2012). The goals of this study are to: (i) determine the pattern of cortical bone growth of the femur (diaphysis); (ii) determine how bone resorption occurs; and (iii) assess ontogenetic and sex tendencies with regard to these processes. We also compare the degree of cortical thickness of *B. suillus* with that of other terrestrial mammals obtained from previous studies. We hypothesize that contrary to observations regarding mineral homeostasis in other mammals, which show an imbalanced (negative) bone modeling throughout ontogeny (Sontag, 1986a; Parfitt, 2010; Montoya-Sanhueza & Chinsamy, 2017), mineral loss in *B. suillus* does not increase considerably with age and they have limited endosteal resorption. Furthermore, since the female skeleton incurs high mineral imbalance during pregnancy and lactation periods (Miller et al., 1986; Tojo et al., 1998), we expect reproductive females of *B. suillus* to show higher levels of intracortical resorption as compared to males, as a result of a generalized mammalian reproductive adaptation. This study further represents the first attempt to determine the skeletal homeostasis and catabolic activity of a mammal with extended lifespan and naturally deprived of vitamin D, thus providing a better understanding of the relationship between bone modeling and physiology in this group of mammals.

## Materials and Methods

### Specimens and osteohistology

Specimens used in this study consisted of a subsample (*n* = 41) of *B. suillus* previously collected at the International Airport of Cape Town, South Africa (for details about specimen collection and animal ethics permits, see Hart et al. (2007) and Montoya-Sanhueza & Chinsamy (2017)). The sample was selected to include a wide range of ontogenetic and reproductive stages (see Table 1 in Montoya-Sanhueza & Chinsamy, 2017). Individuals are grouped as juveniles (age class 2–3), subadults (age class 4–5) and adults (age class 6–9) based on tooth eruption and wear, according to the methodology outlined by Hart et al. (2007). Our sample consists of individuals with the largest body sizes recorded for this species (Bray et al., 2012; Montoya-Sanhueza & Chinsamy, 2017) and previous histological analysis of their limb bones suggests that they represent individuals at the latest stages of somatic growth (Montoya-Sanhueza, 2014; Montoya-Sanhueza & Chinsamy, 2017). Sexually mature females were identified either by having a perforated vagina, been pregnant and/or lactating at time of death. Based on these features, it was apparent that half of the females (*n* = 12) showed at least one copulation event, whilst the rest showed no signs of being sexually active (see Table 1). The reproductive status of the males was determined by considering features such as the size of the seminal vesicle, abdominal or inguinal location of the testes and the presence of sperm. Only four males showed clear signs of reproduction (visible sperm) and one presented inguinal testes (see Table 1 in Montoya-Sanhueza & Chinsamy, 2017).

**Table 1:** Females of *Bathyergus suillus* analyzed in this study.

ID	Ontogenetic stage	Rs	Tr	SeSR	ERC
219	Juvenile	N	+	−	+
307	Juvenile	N	+	−	+
333	Juvenile	N	+	−	+
1373	Juvenile	N	−	−	−
365	Subadult	N	+	−	+
366	Subadult	N	+	−	−
721	Subadult	N	−	+	+
913	Subadult	N	−	−	−
938	Subadult	N	+	−	−
982	Subadult	N	−	−	+
1085	Subadult	Pregnant	−	+	−
1163	Subadult	Pregnant	−	+	+
314	Adult	Pregnant	−	+	−
377	Adult	Perforate	−	+	+
717	Adult	Perforate	+	−	+
911	Adult	N	−	+	−
1138	Adult	Perforate	−	+	+
1144	Adult	Pregnant	−	−	+
1153	Adult	Lactating	−	+	−
1155	Adult	Perforate	−	+	+
1169	Adult	Lactating	−	+	+
1171	Adult	Pregnant	−	+	+
1332	Adult	N	−	+	+
1336	Adult	Perforate	−	+	+

**Notes:**

(i) Reproductive stage (Rs); (ii) presence of trabeculae (Tr) in the medullary cavity; (iii) presence of endosteal secondary remodeling (ESR); and (iv) presence of enlarged resorption cavities (ERC) (based on visual inspection relative to the whole cortical area). No signs of reproduction (N) were observed in juveniles and most subadults. ESR was present in all reproductive females, although also in one non-reproductive subadult female, which also showed ERC. For male data and age classes of both sexes see Montoya-Sanhueza & Chinsamy (2017). Presence (+); Absence (−); No evidence of reproduction (N).

Femora from either the right or left side were extracted and skeletonized for undecalcified histological techniques following Chinsamy & Raath (1992). The bones were cut at the mid-diaphysis (about ∼50% from the proximal articular surface) for the following reasons: (i) this part of the bone is usually considered to be the “neutral region” (Chinsamy-Turan, 2005), i.e., a region with slow relative growth rate due to longitudinal relocation (Enlow, 1963), therefore comprises a section of the bone with scarce growth-related alterations during ontogeny; (ii) represent a good track record of bone formed during earlier ontogenetic stages in *B. suillus* due to scarce endosteal resorption (Chinsamy-Turan, 2005; Montoya-Sanhueza & Chinsamy, 2017); (iii) this part of the diaphysis is considered to be subjected to bending loads (Currey, 1980; Biewener, 1982), and therefore it may show specific ecomorphological and physiological adaptations (Biewener, 1982; Leppänen et al., 2006); (iv) this region is considered to be relevant for predictions of general bone quality (Gluer et al., 1994; Feik, Thomas & Clement, 1997); (v) this region has been shown to be affected by female mammalian reproduction (Vajda et al., 1999; Ross & Sumner, 2017); and (vi) this part of the bone is useful for comparative purposes, since many studies on bone microstructure have focused on the midshaft (Silva & Jepsen, 2013). A total of 43 thin sections of ∼80–100 μm thick were quantitatively and qualitatively analyzed, and high quality photomicrographs were taken using a digital compact camera Canon Power Shot D10 mounted on a Nikon Eclipse E200 Polarizing Microscope. The samples are all housed in the Department of Biological Sciences at the University of Cape Town, South Africa.

### Bone histomorphometry

A total of three static histomorphometric approaches were conducted to determine ontogenetic and sexual differences: (i) diaphyseal histomorphometry (Pinto et al., 2010); (ii) compactness of the bone microanatomy (Montoya-Sanhueza, 2014); and (iii) extent and morphology of intracortical bone resorption (Feik, Thomas & Clement, 1997; Stein et al., 1999). Since studies on the morphology and organization of endosteal cortical bone are still relatively scarce for most mammals, we provide a comprehensive histological description of the (re)modeling processes occurring in these regions, as well as, a quantification of the distribution of resorption cavities (RCs) within different bone matrices in the cortex. The qualitative description of the bone histology and the processes of bone (re)modeling were identified following the terminology of Enlow (1963), Frost (1987), Parfitt et al. (1987), Francillon-Vieillot et al. (1990), de Ricqlès et al. (1991), Chinsamy-Turan (2005) and Parfitt (2010). The concepts of bone modeling and remodeling are explained in Montoya-Sanhueza & Chinsamy (2017).

**Figure 1:**
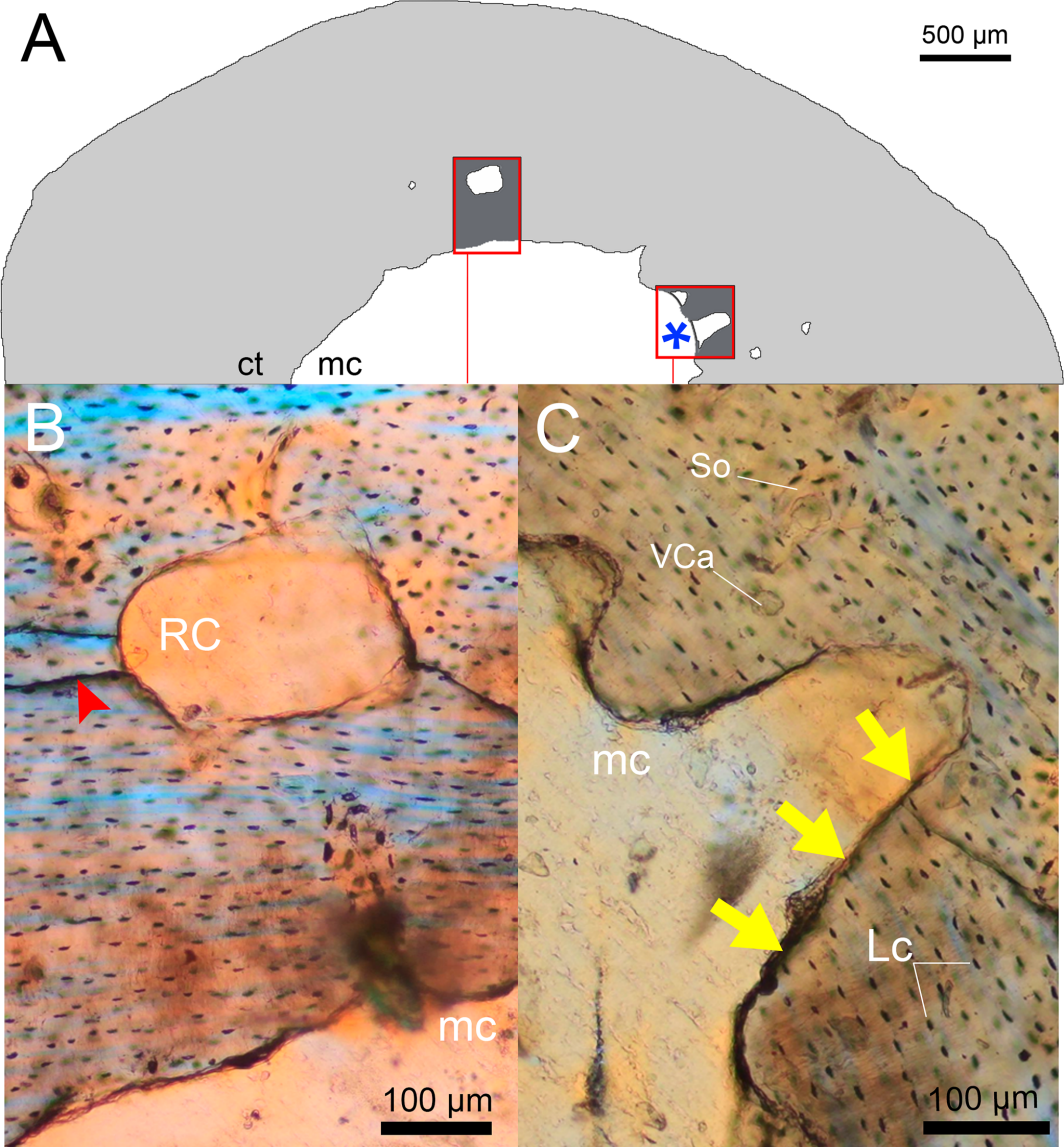
Types of bone resorption in the femoral midshaft of *Bathyergus suillus* (#1050; photographs under polarized light). (A) Half cross section showing the cortical bone (ct) in gray and medullary cavity (mc) and resorption cavities (RCs) as white spaces. The blue asterisk indicates a line drawn to delimit the nearest border between the mc and the RC. (B) Relatively large intracortical RC. Red arrow head indicates lines that result as an artifact after the preparation of the sample. (C) Endosteal resorption forming a bay in the perimedullary region. The resorption surface is uneven and the bone matrices are obliterated due to the osteoclastic activity (yellow arrows). Abbreviations: Lc, osteocyte lacunae; So, secondary osteon; VCa, vascular canal.

High quality images of transverse cross sections of the bones were transformed into binary images by marking bone tissues in gray and resorption spaces in white (Fig. 1A). Since nutrient canals appear to have variable locations and frequency in this species (Montoya-Sanhueza & Chinsamy, 2017), these spaces were considered as solid bone in this study. Binary images were edited in Adobe Photoshop^®^ CS Version 8.0.1 and analyzed in the software Image Pro-Plus version 4.5 (Media Cybernetics, Silver Spring, MD, USA). A total of eight measurements were collected: cross sectional maximum width (Cs.Wi) (= mediolateral diaphyseal width); core or sectional area (C.Ar); medullary cavity area (Me.Ar); endosteal perimeter (Es.Pm); number of RCs per cross section/specimen (n.Rc); and area (Rc.Ar); maximum diameter (Rc.Dm) and roundness of RCs (Rc.Rn). From these measurements, four more parameters were calculated: (i) total resorbed bone area per individual (Tt.Rc.Ar); which is the sum of all resorption cavity areas for each section per individual (Stein et al., 1999); (ii) cortical area (Ct.Ar), which is the difference between C.Ar less Me.Ar and Tt.Rc.Ar (Silva & Jepsen, 2013); (iii) intracortical porosity (Ct.Po), which is Tt.Rc.Ar divided by cortical area (as C.Ar less Me.Ar) (Parfitt et al., 1987; Stein et al., 1999; Dempster et al., 2013); and (iv) the relative cortical area (RCA), which is a dimensionless parameter to quantify ossified area in a section and is obtained from the ratio Ct.Ar/C.Ar (in this case Ct.Ar = C.Ar–Me.Ar). Names, abbreviations and descriptions of the measurements used in this study are presented as a separated document in the Supplementary Files.

The degree of bone compactness was measured using the software Bone Profiler Version 4.5.7 (Girondot & Laurin, 2003). This program has been previously used to assess bone resorption in fish (Deschamps et al., 2009), but mostly to quantify the bone microanatomy (and compactness) of long bones in amniotes and to distinguish morpho-functional features of animals adapted to different lifestyles such as terrestrial, aerial, aquatic or semiaquatic (Buffrénil et al., 2010; Meier et al., 2013). Bone Profiler quantifies the ratio of bone/empty-space in a total cross sectional area by the generation of a grid with 60 radial sectors and 51 concentric segments originated from a longitudinal axis in the center of the section, spreading toward the periphery (Girondot & Laurin, 2003; Buffrénil et al., 2010; Meier et al., 2013). This software renders a rapid quantification of bone distribution around the whole cortex and several other parameters can be obtained from it. A total of five parameters are used in the present study (Girondot & Laurin, 2003): (i) BC, which is a dimensionless score of bone compactness, obtained from the ratio of solid bone tissue area to total section area (equivalent to RCA); (ii) S, a sigmoid curve expressed as the reciprocal of the slope at the inflexion point, which explains the extension of the transitional zone between the medullary cavity and the cortical bone; (iii) P, a distance from the center of the cross section to the transitional zone (without trabeculae), whereby it is proportional to the size of the medullary cavity; (iv) CDI, is the cortico diaphyseal index, which is a body size-independent measure of cortical thickness (i.e., thickness of the cortex divided by the radius of the cross section) (Castanet et al., 2000); and (v) R/t, which is the ratio between the outside radius of the bone wall (R) and its thickness (t), and it represents another body size-independent measure, along with CDI, of the bone thickness and its structural efficiency (Currey & Alexander, 1985; Currey, 2002) (see also document in Supplementary Files).

### Bone resorption

An important goal of this study is to determine catabolic activity in bones, i.e., bone loss that is not related to normal bone growth processes (e.g., angiogenesis and vascularization). Bone resorption occurs when osteoclasts remove both matrix and minerals from bone. This cellular activity results in the formation of Howship’s lacunae and consequently enlarged scalloped surfaces in the cortex (Fig. 1C) (Sissons, Kelman & Marotti, 1984). Bone porosity is one of the physical properties affecting the mechanical properties of the bone (Cowin, 1983; Martin, Burr & Sharkey, 1998), and therefore its study aims to determine changes in whole bone strength and its consequences for fracture risk. However, most studies measuring bone porosity have not made a distinction between the processes forming porous spaces. For this reason, we first distinguished between RCs and other porous spaces in the cortex such as osteocyte lacunae, canaliculi or void space (e.g., blood vessels, osteon lumen, Haversian canals and nutrient arteries) (Parfitt et al., 1987; Dempster et al., 2013). Void space is an inherent aspect of bone microstructure and is, most probably, already adapted to not considerably compromise bone strength and mechanical function (de Margerie, Tafforeau & Rakotomanana, 2006). Although previous studies have used different concepts of cortical porosity (Chinsamy, 1993; Stein et al., 1999; Thomas, Feik & Clement, 2005; Mukherjee, Ray & Sengupta, 2010), here we consider intracortical porosity as all the cavities undergoing visible osteoclastic resorption, regardless of size, shape and location. Consequently, identification of RCs was made under a microscope by visual inspection of cavities with uneven borders.

**Figure 2:**
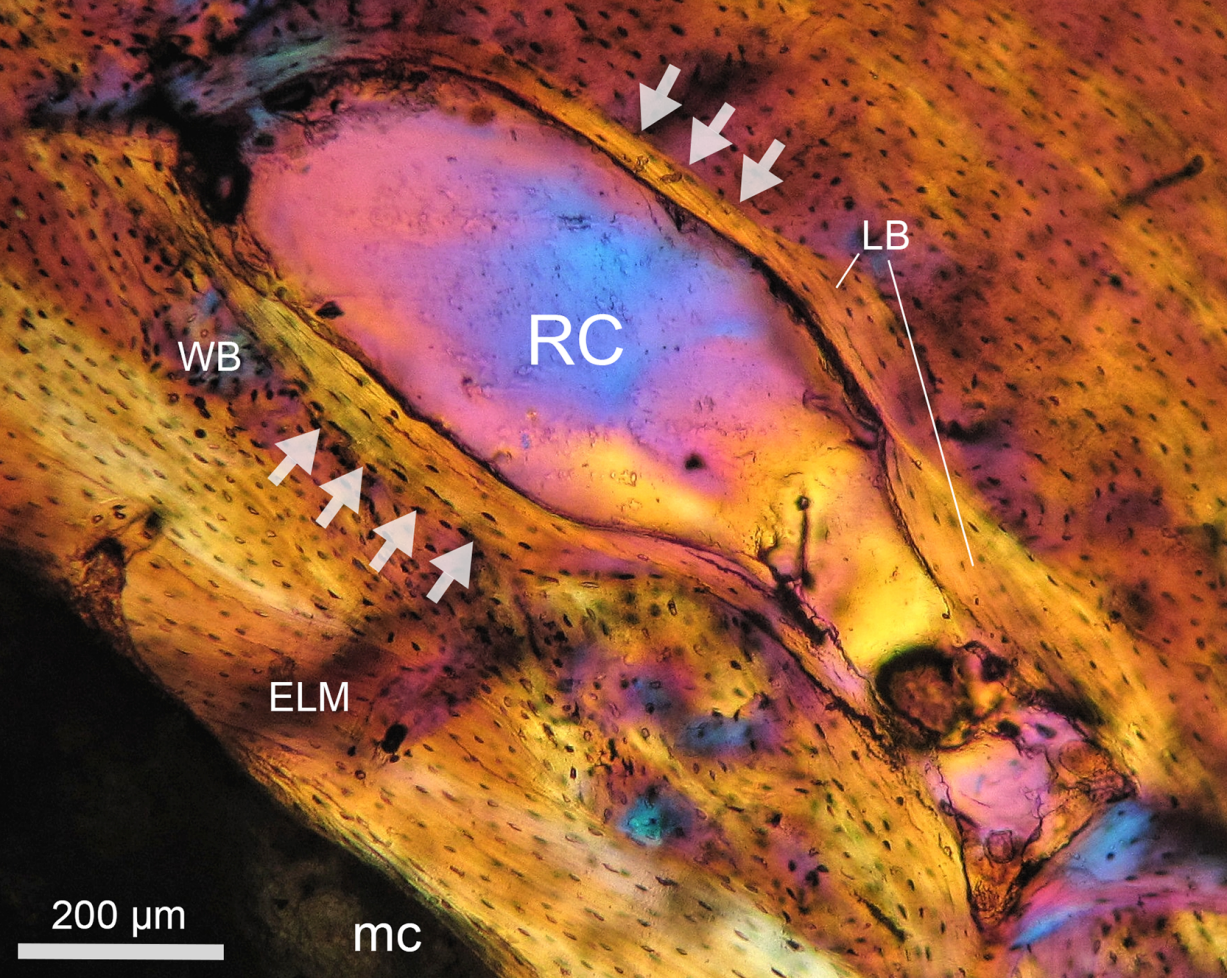
Resorption cavity (RC) undergoing secondary bone remodeling in *Bathyergus suillus* (#S2, see Montoya-Sanhueza (2014); photograph under polarized light). A thin layer of lamellar bone (LB) is deposited internally around the RC, which is delimited by a reversal line (arrows). The RC occurs within woven bone (WB). Endosteal lamellar bone (ELB) underlies the WB matrix and lines the medullary cavity (mc).

It is important to consider that bone resorption can occur either within the cortex (intracortical resorption) and/or along the endosteal/periosteal surfaces (endosteal/periosteal resorption). Intracortical resorption is identifiable by the occurrence of typical RCs (Fig. 1B), although some of them can be open to the medullary cavity due to either endosteal or extended intracortical resorption, thus forming enlarged bays of bone resorption located along the inner or perimedullary margins of the cortex (Fig. 1C) (Keshawarz & Recker, 1984). In this study, some bays of endosteal resorption were considered as intracortical resorption, since they extended toward the cortical bone (Fig. 1C), and additional slight manual polishing of these regions indicated that these corresponded to RCs, i.e., totally integrated within the cortex. In these cases, a thin line was digitally drawn on images to delimit the resorbed area to the approximate perimeter of the medullary cavity (Fig. 1A). Significantly large RCs undergoing secondary reconstruction, i.e., with some centripetal deposition of lamellar bone (Fig. 2), were also included in the analysis since they are considered to represent clear evidence of previous catabolic activity (Parfitt, 1984; Goldman et al., 2009; Zebaze et al., 2010; Lerebours et al., 2015).

### Statistical analysis

Two-way ANOVA were performed to assess the effects of sex and ontogeny in histomorphometric parameters. For determination of sexual dimorphism in parameters related to bone resorption (i.e., n.Rc, Rc.Ar, Rc.Wi, Rc.Pm, Rc.Rn, Tt.Rc.Ar and Ct.Po), only reproductive (adults) and potentially reproductive individuals (subadults) (based on Hart et al., 2007) were analyzed. Scheffé post hoc test was used to correct potential type I error in the two-way ANOVA analyses. To assess sex differences in shape and size of RCs (i.e., Rc.Ar, Rc.Wi, Rc.Pm, Rc.Rn), the data were analyzed with a linear mixed-effects model, with individuals as the random factor (repeated measures) and sex as the fixed one, since one specimen can have more than one RC (West, Welch & Galecki, 2015). Data are presented as Mean ± Standard Deviation (SD).

Additionally, linear regressions (ordinary least square—LS) of all microstructural traits were fitted against femoral length to assess ontogenetic trends and sexual dimorphism. Ontogenetic trends were assessed by two separate models, one for each sex, while sex differences were assessed by slope comparison (interaction term between sexes). We selected femoral length as a proxy of the individual’s size (rather than body mass and body length) (Table 2) due to its convenience for further comparative analyses with modern and fossil (archaeological and paleontological) data. Femoral length also scales isometrically with body mass (LS, slope: 0.305; confidence intervals (CI): 0.267–0.334; *n* = 41) and body length (LS, slope: 1.013; CI: 0.8982–1.081; *n* = 42), which indicates that this element represents a good estimator of general body size and skeletal maturity in *B. suillus*. Males and females also showed similar isometric patterns. A 95% CI was used in all statistical analyses. Box plots presented in this study show the following legend: (1) the dark horizontal line in the middle of the boxes is the median; (2) the boxes indicate the 25^th^ (bottom) and 75^th^ (top) percentile of the cases; (3) the T-bars that extend from the boxes are the whiskers, which represent 1.5 times the height of the box when data is available for bottom and top boxes; however, if no value exist in any of these ranges (bottom and/or top), it will represent the minimum or maximum values, respectively; (4) the points (hollow circles) are outliers, and are defined as values that do not fall within the whiskers. Width of the boxes indicates sample size. Statistical analyses were performed in IBM SPSS version 24 (IBM, Armonk, NY, USA) Statistical Package for Social Sciences.

**Table 2:** Body size parameters (Mean ± SD) of *Bathyergus suillus*; and results of the two-way ANOVA showing significance levels for differences in ontogeny, sex and their interaction.

	Juveniles		Subadults		Adults		Two-way ANOVA (*p*)									Scheffé test
	Male (*n* = 2)	Female (*n* = 3)	Male (*n* = 8)	Female (*n* = 8)	Male (*n* = 7)	Female (*n* = 12)	df	F	*p*-Sex	df	F	*p*-Age	df	F	*p*-Sex × Age	*p* < 0.001
BM (g)	533 ± 91.92	318 ± 135.47	843.25 ± 225.81	623.25 ± 178.06	1474.57 ± 186.92	991.67 ± 203.93	1	16.94	<0.001	2	45.62	<0.001	2	2.19	0.127	J < S < A
BL (mm)	260 ± 7.07	218.33 ± 22.54	308.13 ± 13.07	275.38 ± 13.07	366 ± 12.79	317.75 ± 9.41	1	72.14	<0.001	2	148.35	<0.001	2	1.59	0.220	J < S < A
FL (mm)	34.6 ± 0.14	31.33 ± 1.76	43.18 ± 3.88	39.41 ± 2.49	52.14 ± 2.45	46.29 ± 2.58	1	16.73	<0.001	2	77.04	<0.001	2	0.82	0.449	J < S < A

**Notes:**

Abbreviations: body mass (BM), body length (BL) and femoral length (FL)

## Results

### Femoral length and body size

A total of 41 specimens comprising eight relative age classes (2–9) were analyzed. Femoral length is highly correlated with body mass (BM) (*R*^2^ = 0.87) and body length (BL) (*R*^2^ = 0.88), whereas BM also correlates well with BL (*R*^2^ = 0.82). Significant ontogenetic differences and a male-biased pattern of sexual dimorphism were evidenced by two-way ANOVA analyses (Table 2). Based on cranial morphometric analysis, Hart et al. (2007) reported similar results for individuals between age classes 6 and 9, although they did not assess differences in femoral length. It is expected that unlike social molerats (Bennett & Faulkes, 2000), where there is obvious social ranks, there would not be additional effects on body mass and body length in *B. suillus* (Hart et al., 2007).

### Diaphyseal changes with age and sex

The femoral midshaft is generally composed of cortical bone with an open medullary cavity (Fig. 1A), although 29% of the females, especially juveniles and subadults showed short thick trabeculae (Table 1), while only one male presented these features (subadult, #1339). A more detailed description of the microstructural changes occurring in the femoral midshaft of *B. suillus* is provided in Montoya-Sanhueza & Chinsamy (2017).

**Figure 3:**
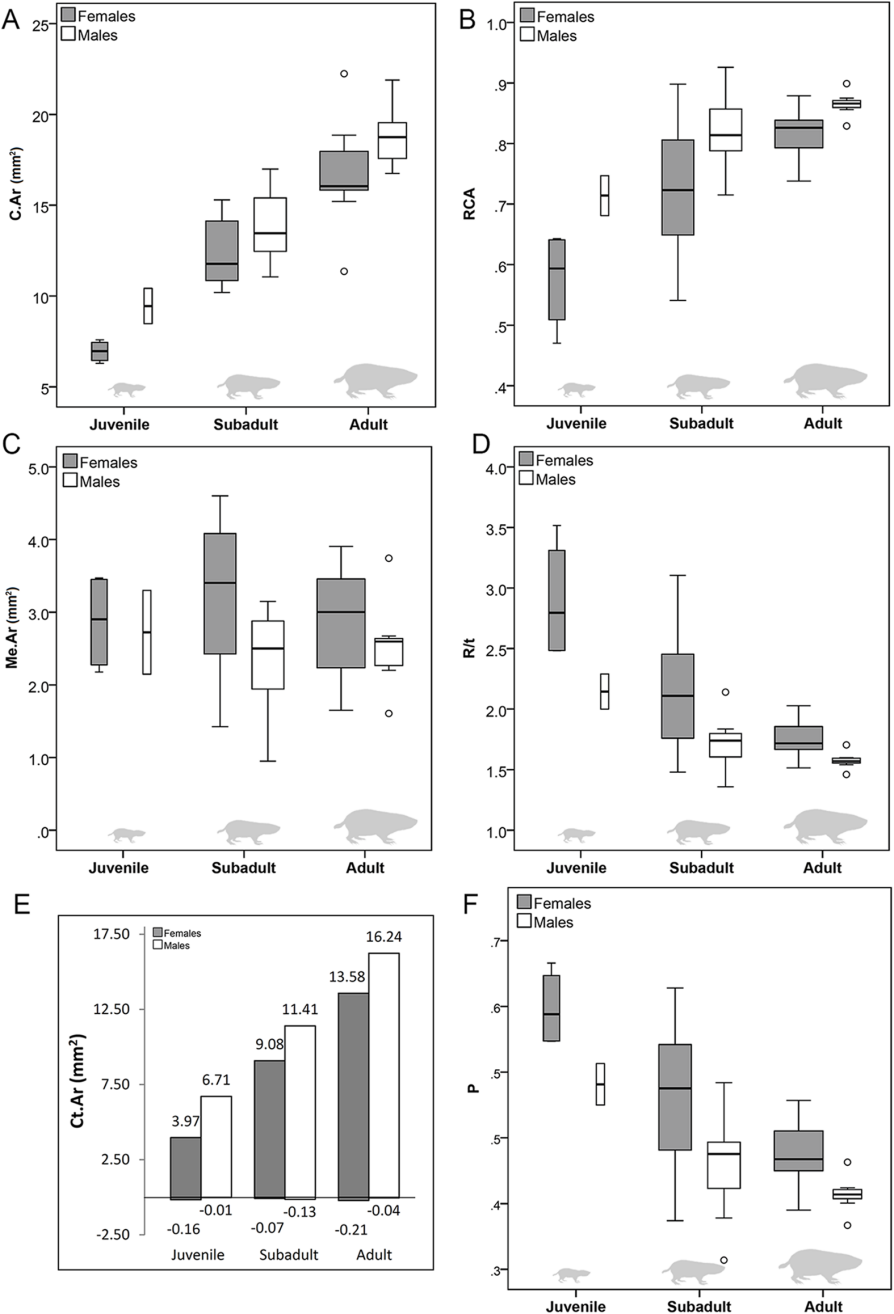
Ontogeny of histomorphometric traits in *Bathyergus suillus*. Box and whisker plots showing: (A) cross sectional area (C.Ar); (B) relative cortical area (RCA); (C) medullary cavity area (Me.Ar); (D) the ratio between outer radius of the bone and its wall thickness (R/t); (E) bar graph showing mean cortical bone formation (gain/expansion) and resorption (loss) during ontogeny; and (F) P parameter showing the distance from the center of the medullary cavity to the transitional cortical zone (see text). See box plot legend in text.

The two-way ANOVA showed that most of the diaphyseal traits (Cs.Wi, C.Ar, Ct.Ar and RCA) differ with age and sex, although Es.Pm only differs with sex (Table 3). These data show significant increases in periosteal bone formation with aging in both sexes (Figs. 3A and 3B). In general, males augmented their cortical area (Ct.Ar) by 69% from juvenile to subadults, and by 42% from subadult to adult stages, while females augmented their Ct.Ar by 129% and 49%, respectively (Table 3; Figs. 3B and 3E). To reach adult size, male juveniles increased their Ct.Ar by 142% and female juveniles by 242%. Thus, it is apparent that most of the growth expansion occurs during the transition from juvenile to subadults. At juvenile stages, females show lesser Ct.Ar as compared to males, but they show a quick increase in cortical expansion afterwards. The latter is illustrated in the regression analysis, where females showed a steeper slope in RCA (see also BC and R/t below) as compared to males, although the rest of the traits did not show any significant difference between sexes (Table 4). An interesting trend was found in the size of the medullary cavity (Me.Ar), which does not change significantly during ontogeny, although this parameter varies among individuals (Table 3; Fig. 3C). Despite the fact that there are not significant differences between the sexes for this trait, females tend to have slightly larger medullary cavities, as well as higher values of Es.Pm (*p* = 0.04) when compared to males of the same age (Table 3). These two last parameters may indicate a higher degree of endosteal resorption in females as compared to males, although there is not a considerably strong signal for Me.Ar to be statistically significant. The analysis of diaphyseal traits in *B. suillus* indicate a generalized unbalanced (positive) modeling activity, with high periosteal bone formation and scarce resorption at perimedullary regions of the cortex (Fig. 3E).

**Table 3:** Descriptive femoral histomorphometry (Mean ± SD) of *Bathyergus suillus* and results of the two-way ANOVA showing tendencies during ontogeny and between sexes.

	Juveniles		Subadults		Adults		Sex			Age			Sex* Age			Scheffé test
	Male (*n* = 2)	Female (*n* = 4)	Male (*n* = 8)	Female (*n* = 8)	Male (*n* = 7)	Female (*n* = 12)	df	F	*p*	df	F	*p*	df	F	*p*	Age
Cs.Wi (mm)	3.97 ± 0.23	3.54 ± 0.18	5.06 ± 0.49	4.79 ± 0.51	6.06 ± 0.29	5.68 ± 0.58	1	4.200	**0.048**	2	44.899	**<0.001**	2	0.079	0.924	J < S < A
C.Ar (mm^2^)	9.45 ± 1.37	6.95 ± 0.60	13.83 ± 1.98	12.37 ± 1.91	18.81 ± 1.73	16.64 ± 2.57	1	7.330	**0.010**	2	50.968	**<0.001**	2	0.186	0.831	J < S < A
Ct.Ar (mm^2^)	6.71 ± 0.54	3.97 ± 0.36	11.41 ± 2.25	9.08 ± 2.52	16.24 ± 1.14	13.58 ± 2.23	1	11.931	**0.001**	2	52.809	**<0.001**	2	0.037	0.964	J < S < A
Me.Ar (mm^2^)	2.72 ± 0.82	2.86 ± 0.68	2.34 ± 0.72	3.23 ± 1.10	2.54 ± 0.65	2.89 ± 0.73	1	2.407	0.130	2	0.046	0.955	2	0.670	0.518	J = S = A
Es.Pm (mm)	6.00 ± 0.80	6.44 ± 0.87	5.71 ± 0.97	6.93 ± 1.07	6.20 ± 0.63	6.66 ± 0.86	1	4.654	**0.038**	2	0.142	0.868	2	0.862	0.431	J = S = A
RCA	0.71 ± 0.05	0.58 ± 0.08	0.82 ± 0.07	0.72 ± 0.11	0.87 ± 0.02	0.82 ± 0.04	1	14.167	**0.001**	2	17.587	**<0.001**	2	1.071	0.354	J < S < A
BC	0.71 ± 0.06	0.57 ± 0.08	0.82 ± 0.07	0.72 ± 0.12	0.86 ± 0.02	0.81 ± 0.04	1	14.153	**0.001**	2	17.100	**<0.001**	2	1.029	0.368	J < S < A
CDI	0.47 ± 0.04	0.35 ± 0.06	0.59 ± 0.08	0.49 ± 0.11	0.64 ± 0.03	0.57 ± 0.05	1	12.504	**0.001**	2	15.569	**<0.001**	2	0.485	0.620	J < (S = A)
R/t	2.15 ± 0.21	2.90 ± 0.51	1.72 ± 0.23	2.15 ± 0.52	1.58 ± 0.07	1.75 ± 0.15	1	15.917	**<0.001**	2	16.141	**<0.001**	2	1.971	0.154	J > (S = A)
S	0.02 ± 0.01	0.03 ± 0.01	0.02 ± 0.01	0.02 ± 0.01	0.02 ± 0.01	0.03 ± 0.01	1	7.343	**0.010**	2	2.392	0.106	2	1.734	0.191	J = (S ≠ A)
P	0.53 ± 0.04	0.65 ± 0.06	0.41 ± 0.08	0.51 ± 0.11	0.36 ± 0.03	0.43 ± 0.05	1	12.504	**0.001**	2	15.586	**<0.001**	2	0.484	0.621	J > (S = A)
n.Rc	2.5 ± 2.12	4 ± 4.36*	12.2 ± 7.16†	3.33 ± 1.75‡	4.17 ± 3.19‡	3.7 ± 3.97ᵻ	1	2.388	0.134	2	3.258	0.055	2	4.054	**0.029**	J = S = A
Tt.Rc.Ar (mm^2^)	0.01 ± 0.02	0.16 ± 0.21*	0.13 ± 0.08†	0.07 ± 0.05‡	0.04 ± 0.05‡	0.21 ± 0.17ᵻ	1	3.009	0.095	2	0.179	0.837	2	2.831	0.077	J = S = A
Ct.Po (%)	0.19 ± 0.23	4.55 ± 6.14*	1.24 ± 0.83†	0.72 ± 0.60‡	0.24 ± 0.32‡	1.47 ± 1.21ᵻ	1	5.278	**0.030**	2	1.115	0.343	2	2.715	0.085	J = S = A

**Notes:**

Abbreviations: C.Ar, core or sectional area; Cs.Wi, cross sectional maximum width; Ct.Ar, cortical area; Ct.Po, intracortical porosity; Es.Pm, endosteal perimeter; Me.Ar, medullary cavity area; n.Rc, number of resorption cavities per cross section/specimen; RCA, the relative cortical area; Tt.Rc.Ar, total resorbed bone area per individual.

Significant *p*-values in bold.

* (*n* = 3).

† (*n* = 5).

‡ (*n* = 6).

ᵻ (*n* = 10).

**Table 4:** Regression analysis showing the linear relationship between femoral length (FL) and histomorphometric parameters of *Bathyergus suillus*.

	Regression analysis (Independent models for each sex)											
	Males					Females					Slope comparison	
	*R*^2^	*Y*-Intercept	Slope	CI.min	CI.max	*R*^2^	*Y*-Intercept	Slope	CI.min	CI.max	*t*-test	*p*
Cs.Wi vs. FL	0.79	0.5135	0.1053	0.0796	0.1298	0.82	−0.4639	0.1328	0.1044	0.1574	1.4120	0.1660
C.Ar vs. FL	0.89	−8.2947	0.5158	0.4218	0.6108	0.84	−11.1470	0.5984	0.4685	0.7041	1.0720	0.2910
Ct.Ar vs. FL	0.92	−11.1170	0.5224	0.4522	0.6008	0.85	−14.4350	0.6024	0.4894	0.6971	1.1120	0.2730
Me.Ar vs. FL	0.00	2.6300	−0.0036	−0.0523	0.0416	0.01	3.3719	−0.0091	−0.0549	0.0380	−0.1400	0.8890
Es.Pm vs. FL	0.01	5.3291	0.0134	−0.0375	0.0615	0.00	6.6220	0.0022	−0.0486	0.0519	−0.2530	0.8010
RCA vs. FL	0.57	0.4859	0.0074	0.0043	0.0107	0.64	0.1444	0.0145	0.0102	0.0189	2.3360	**0.0250**
BC vs. FL	0.56	0.4801	0.0075	0.0043	0.0109	0.64	0.1390	0.0145	0.0106	0.0191	2.2990	**0.0270**
CDI vs. FL	0.49	0.2247	0.0081	0.0047	0.0123	0.61	−0.0479	0.0134	0.0098	0.0173	1.6690	0.1040
R/t vs. FL	0.53	2.9221	−0.0263	−0.0408	−0.0140	0.65	4.9300	−0.0689	−0.0948	−0.0468	−3.0900	**0.0040**
S vs. FL	0.00	0.0221	−0.0000507	−0.0005	0.0004	0.02	0.0184	0.0002	−0.0005	0.0009	0.6360	0.5290
P vs. FL	0.49	0.7750	−0.0081	0.0123	−0.0044	0.61	1.0479	−0.0135	−0.0174	−0.0096	−1.6710	0.1030
n.Rc vs. FL	0.08	18.3200	−0.2500	−0.5095	0.4425	0.01	1.4332	0.0526	−0.2954	0.3692	1.1440	0.2620
Tt.Rc.Ar vs. FL	0.07	0.1983	−0.0028	−0.0078	0.0057	0.04	−0.0604	0.0052	−0.0066	0.0174	1.1200	0.2720
Ct.Po vs. FL	0.13	0.0232	−0.0003	−0.0008	0.0004	0.12	0.0736	−0.0013	−0.0034	0.0011	−1.0950	0.2830

**Notes:**

Confidence intervals (CI) are showed for male and female regression slopes. Significant differences in slope values between sexes are indicated in bold. See Table 3 for Abbreviations.

The parameters of bone compactness showed that BC, CDI, R/t and P also differ significantly with age and sex, while the parameter S differs only with sex (Table 3; Figs. 3D and 3F). Nevertheless, CDI, R/t and P of juveniles are different from subadults and adults, and these later are not statistically differentiated after post hoc testing (Table 3). Contrarily, post hoc testing showed that the S parameter of juveniles do not differ from subadults and adults, although these two later are different between each other (Table 3). The parameter BC showed similar values as compared to RCA, as both of them reflect proportion of bone within the cross section (Table 3). Information about the interrelationship between periosteal expansion and medullary cavity size was obtained from the parameters CDI and R/t, which showed a relative “decrease” in the medullary cavity size with respect to the whole cross section during ontogeny (Table 3; Fig. 3D). Considering that the medullary cavity maintains its size during ontogeny (Table 3; Fig. 3B), it is inferred that most of the cortical thickening is reached by periosteal expansion, rather than by endosteal apposition (i.e., medullary cavity occlusion). The P parameter also indicated sex differences, with females having higher *P*-values than males, which indicate higher relative distances between the center of the cross section and the transitional zone between bone surfaces (Table 3; Fig. 3F). This indicates larger medullary cavity sizes in females. Females also have significantly higher parameter S (*p* = 0.02) (Table 3) as compared to males. This parameter indicates the degree of structural bone organization in the transitional zone, whereby females show higher degrees of endosteal and subendosteal resorption as well. Regression analysis showed significant differences in BC and R/t, with females showing significantly high slope values (*p* = 0.004) (Table 4), indicative of quicker cortical expansion as compared to males.

### Intracortical porosity

A total of three analyses were performed to assess different aspects of intracortical porosity: (i) the first to measure the degree of cortical porosity in the whole cross section; (ii) the second to quantify the incidence of RCs in different bone matrices; and (iii) the third to determine histomorphometric differences of individual RCs between sexes.

**Figure 4:**
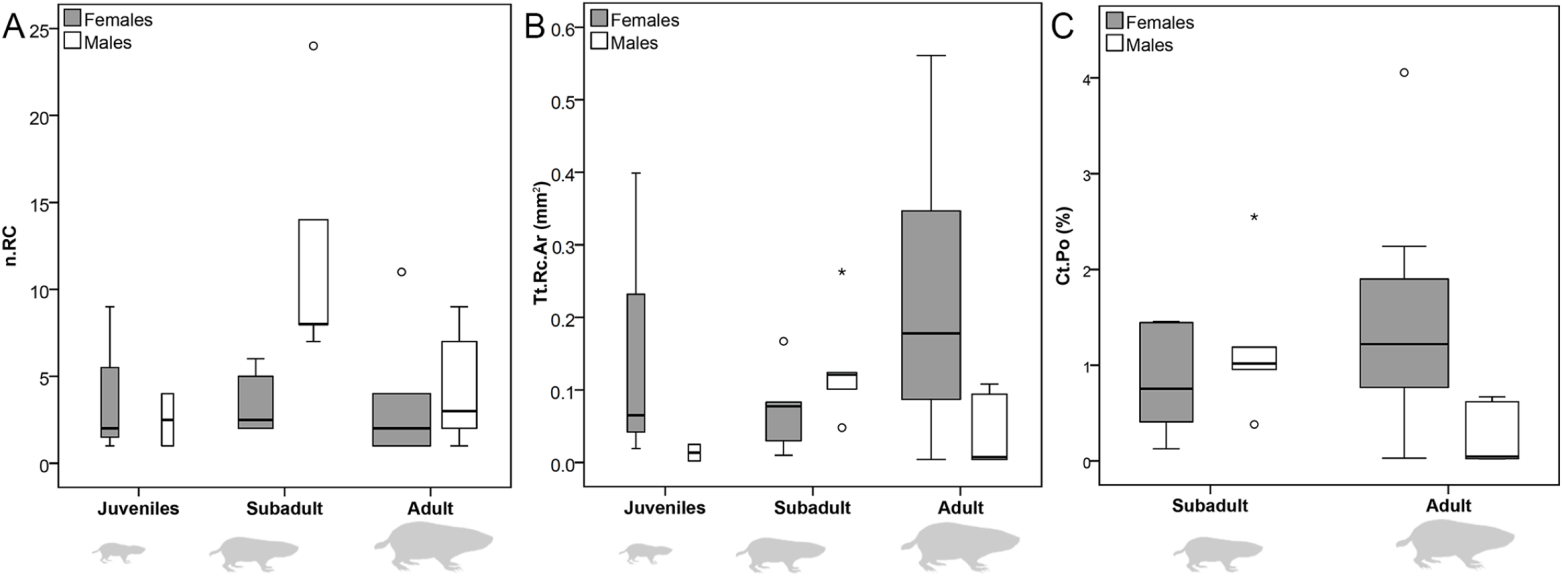
Box and whisker plots showing ontogenetic trends of *Bathyergus suillus*. (A) number of resorption cavities (n.Rc); (B) total resorbed bone are (Tt.Rc.Ar); and (C) cortical porosity (Ct.Po) in subadult and adult stages only.

A total of 32 individuals (78%) showed RCs, with females showing a slightly higher incidence of them (♀ = 79.2%; ♂ = 76.5%) (Table 1). No statistically significant differences in the number of RCs (n.Rc) and total resorbed bone area (Tt.Rc.Ar) per individual were found when analyzed by age or sex, although intracortical porosity (Ct.Po) differs with sex (Table 3; Fig. 4). Females have higher Ct.Po values (*p* = 0.03) and hence resorb more bone, relative to their cortical bone area, than males (Fig. 4C). Nevertheless, it is important to highlight that the levels of bone loss in both sexes are overall quite low when compared to the levels of bone gained during ontogeny (Fig. 3E). Despite the non-significant differences in n.Rc and Tt.Rc.Ar, males show a tendency to have higher n.Rc than females, especially non-juveniles (Fig. 4A). On the other hand, females tend to show greater Tt.Rc.Ar as compared to males, except in subadult stages (Fig. 4B). When all age classes are analyzed by linear regression, CDMs do not show any specific ontogenetic pattern in these traits (Table 4), although the two-way ANOVA showed a significant interaction for the two factors and n.Rc (Table 3).

**Figure 5:**
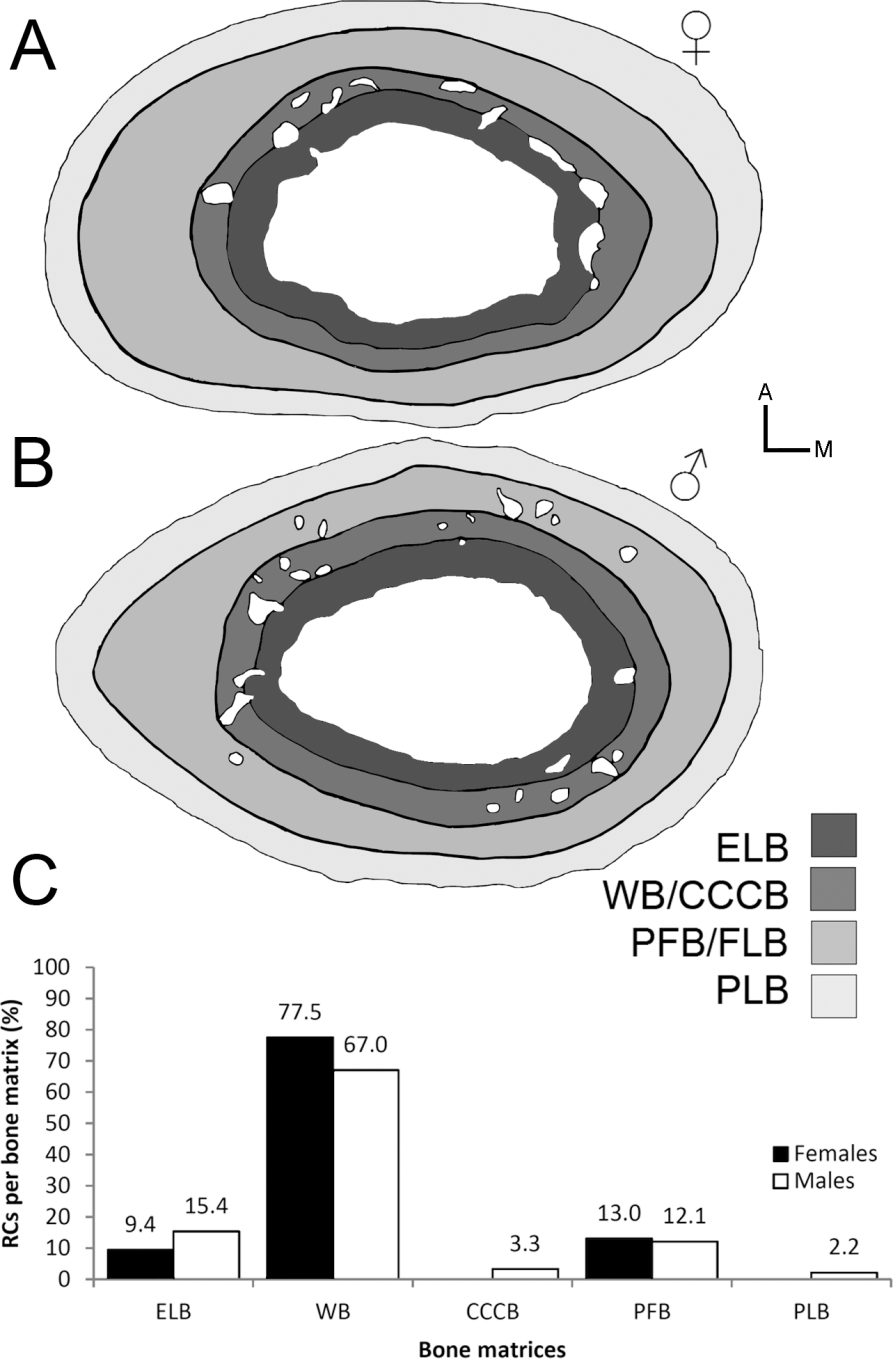
Distribution of resorption cavities (RCs) within the cortex of *Bathyergus suillus*. (A) Cross section of a female (#1169); (B) cross section of a male (#765); (C) bar graph showing the distribution of RCs in different bone tissues (see data in Table 5). Abbreviations: CCCB, compact coarse cancellous; ELB, endosteal; PLB, periosteal lamellar; WB, woven bone; FLB, fibrolamellar and PFB, parallel fibered bone tissues.

**Figure 6:**
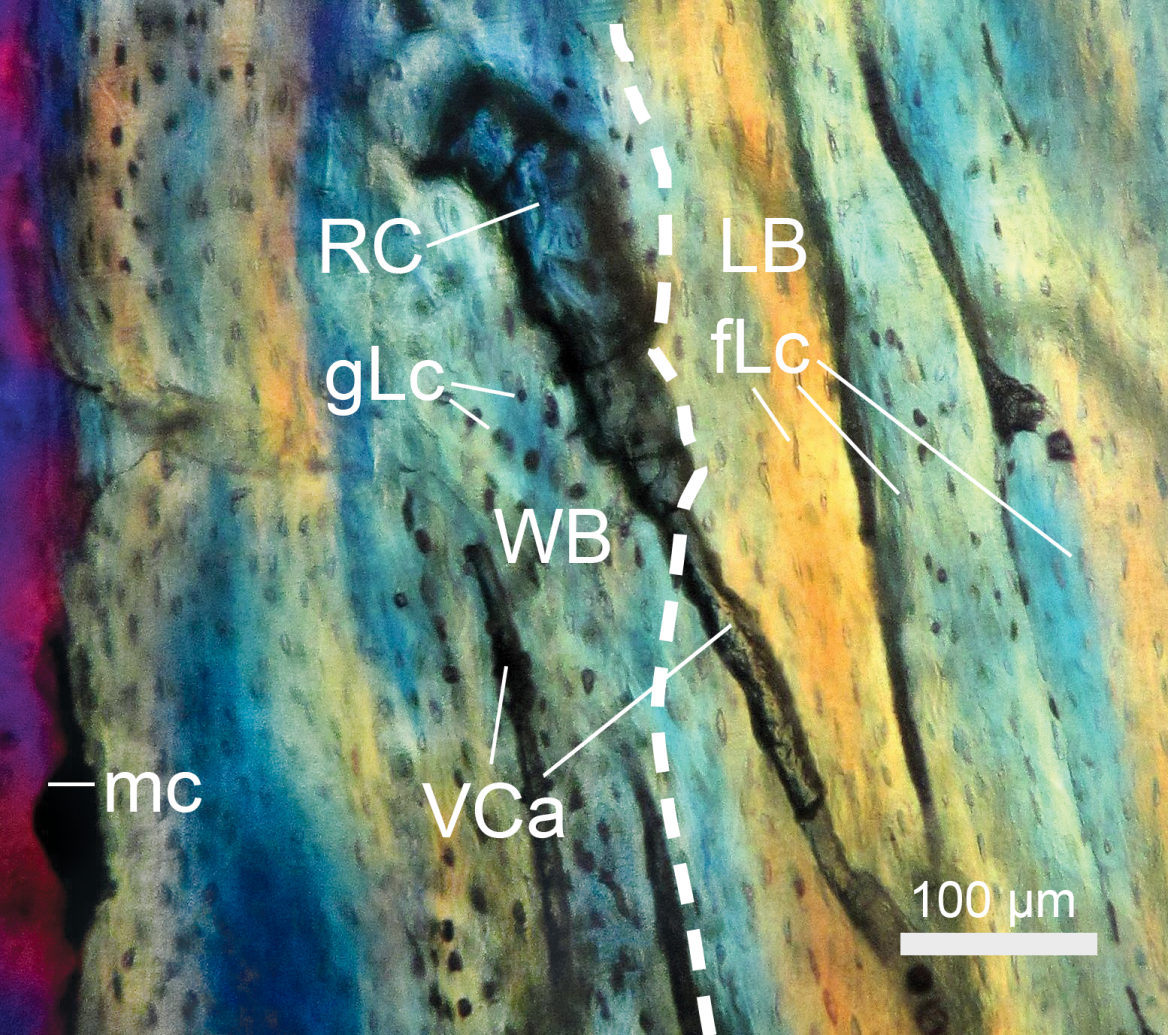
Longitudinal section of a femoral midshaft showing a resorption cavity (RC) within a woven bone (WB) matrix (#S1, see Montoya-Sanhueza (2014); photograph under polarized light). The dashed line demarcates the limit between woven bone (WB) and lamellar bone (LB). Globular (gLc) and flattened osteocyte lacunae (fLc) occur in the different tissue types. (mc) indicates the medullary cavity.

Resorption cavities were present within the entire cortex regardless of sex (Figs. 5A and 5B), but occur mostly in endosteal and subendosteal regions (∼86.9% in females; ∼82.4% in males) (Fig. 5C; Table 5). The rest of the RCs were distributed toward the outer surface of the cortex, where fibrolamellar, parallel-fibered and periosteal lamellar bone tissues constitute the predominant matrices (Fig. 5; Table 5). It is interesting to note that most of the RCs are developed in a woven bone matrix (∼73.87%), especially in females (Fig. 5C). This specificity for resorbing woven bone was also observed when longitudinal cross sections were observed along a vascular canal with resorptive activity, i.e., the canal crossed several layers of bone tissue, but it develops a RC only in the region of woven bone (Fig. 6).

**Table 5:** Incidence of resorption cavities (RCs) in different bone matrices in *Bathyergus suillus*.

Bone matrix	Juveniles		Subadults		Adults		Total RCs
	Males	Females	Males	Females	Males	Females	
ELB	0	2	8	3	6	0.5	19.5
WB	5	6	42	15	14	32.5	114.5
CCCB	0	0	1	0	2	0	3
PFB	0	0	8	2	3	3	16
PLB	0	0	2	0	0	0	2
Total RCs	5	8	61	20	25	36	155

**Note:**

See Fig. 5 for Abbreviations.

**Figure 7:**
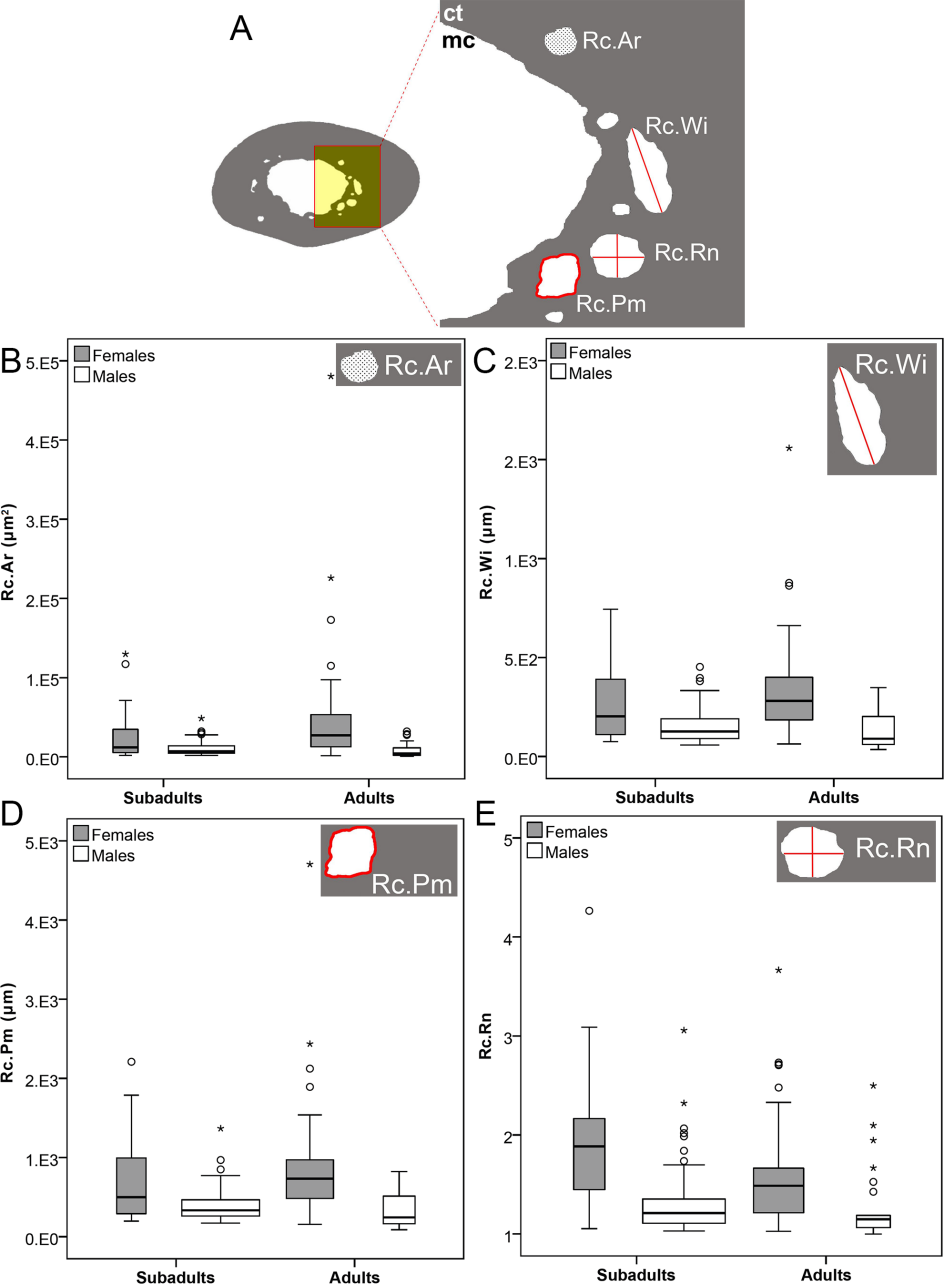
Bone histomorphometry of resorption cavities and sex differences in *Bathyergus suillus*. (A) Graph showing histomorphometric parameters for characterization of resorption cavities; (B) box and whisker plots of resorption cavity area (Rc.Ar); (C) width (Rc.Wi); (D) perimeter (Rc.Pm); and (E) roundness (Rc.Rn). Abbreviations: ct, cortical bone; mc, medullary cavity.

**Figure 8:**
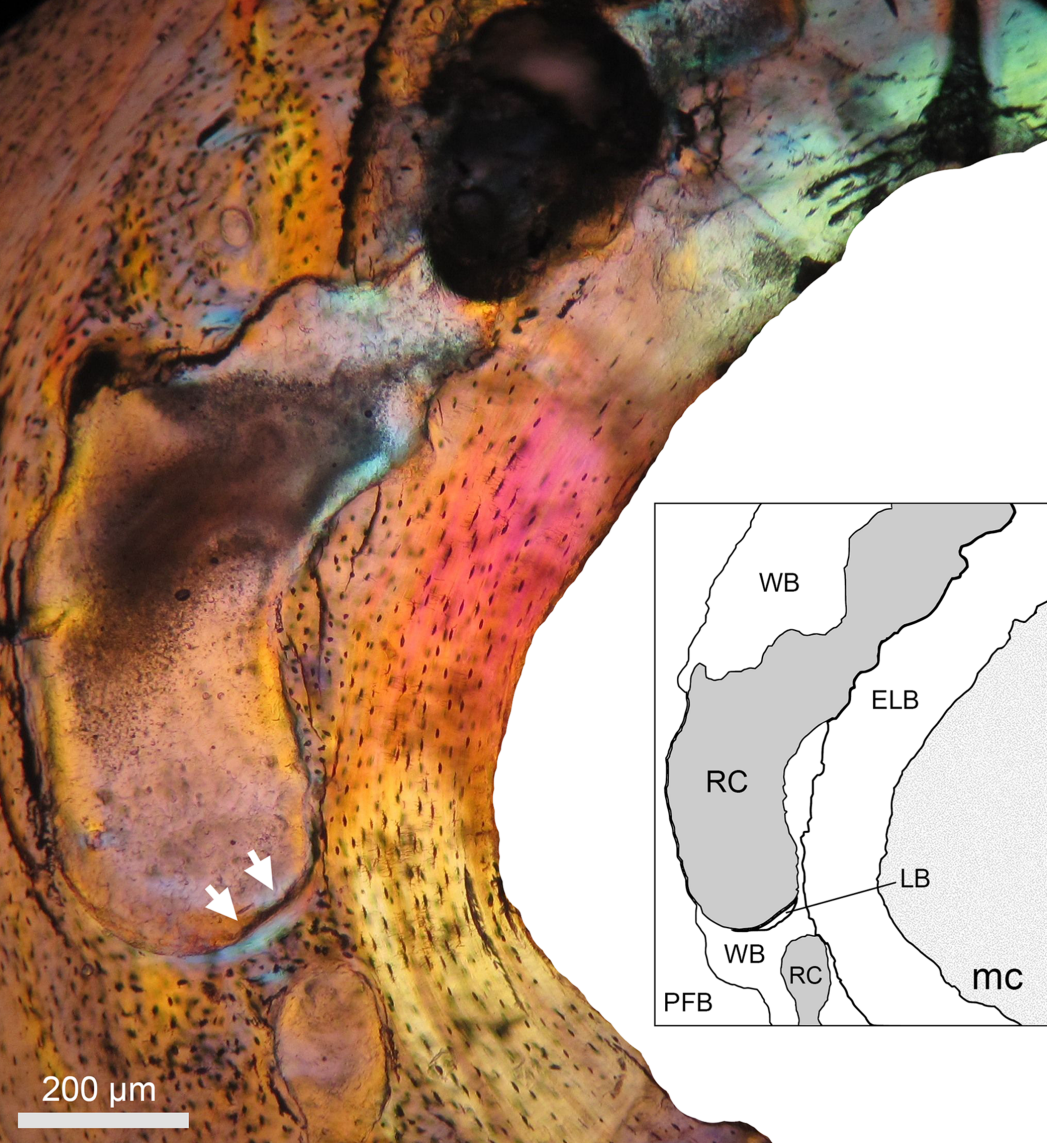
An enlarged resorption cavity (RC) of a female (#1336) extending circumferentially in the subendosteal region of the cortex (photograph under polarized light). Enlarged RCs usually extend within a band of woven bone (WB) and are surrounded by endosteal lamellar bone (ELB) and parallel fibered bone (PFB). Some centripetally formed lamellar bone (LB) is observed in the lower part of the RC (white arrows). Abbreviations: mc, medullary cavity.

For the assessment of histomorphometric differences between sexes, only reproductive (adults) and potentially reproductive (subadult) individuals were analyzed. The morphology of 146 RCs was assessed, and showed that females have statistically significant higher values in all traits (Rc.Ar, Rc.Wi, Rc.Pm and Rc.Rn) (Table 6; Figs. 7B–7E). The maximum RC size recorded in males was ∼48,684 μm^2^, which is similar to the mean Rc.Ar of females (∼43,947, μm^2^) (Table 6). The maximum pore size of females was around 90% larger than the maximum pore size of males (Table 6). Females also showed RCs of irregular shape (Fig. 7, also Fig. 5A), as indicated by Rc.Wi and Rc.Rn values (Table 6; Figs. 7C and 7E), whilst males had smaller and circumferential RCs (Figs. 7C and 7E). Consequently, females, apart from having higher amount of intracortical porosity (Table 3; Fig. 4), they also have larger and non-circular RCs when compared to males. The enlargement of RCs in females showed a particular pattern of expansion around the medullary cavity (Fig. 8; also see Figs. 2 and 5A), which is documented in detail in the next section.

**Table 6:** Linear Mixed-Effects (LME) analysis of *Bathyergus suillus* showing sex differences in the morphology (Mean ± SD) of the resorption cavities (RC); resorption cavity area (Rc.Ar), width (Rc.Wi), perimeter (Rc.Pm) and roundness (Rc.Rn) of RCs.

Measurements	Male (*n* of RCs = 86)				Female (*n* of RCs = 60)				Repeated measures	
	Mean ± SD	Min	Max	CV	Mean ± SD	Min	Max	CV	F	*p*
Rc.Ar (μm^2^)	10264.26 ± 9574.28	815.12	48684.93	93.3	43947.51 ± 71577.05	1585.97	480571.70	162.9	8.45	0.011
Rc.Wi (μm)	143.19 ± 87.19	35.21	452.88	60.9	324.09 ± 262.44	63.45	1558.26	81.0	17.97	0.001
Rc.Pm (μm)	383.00 ± 222.48	88.47	1367.64	58.1	852.63 ± 733.63	157.74	4704.92	86.0	16.31	0.001
Rc.Rn	1.32 ± 0.37	1	3.06	28.0	1.74 ± 0.68	1.03	4.26	39.3	17.47	0.001

**Note:**

Data also show minimum (min) and maximum (max) values observed and coefficient of variation (CV).

### Endosteal bone histology and bone remodeling

Here we provide a detailed description of the secondary reconstruction observed in the femur of CDMs. The inner regions of the cortex are composed mostly of endosteal lamellar, woven bone and compact coarse cancellous bone tissues (Figs. 5A and 5B). In general, the layer of endosteal lamellar bone increases its thickness during ontogeny, while woven bone seems to reduce its predominance. The latter bone tissue is often closer to other bone matrices such as compact coarse cancellous bone, or as being part of fibrolamellar bone toward the outer regions of the lateral side of the cortex (Fig. 5).

**Figure 9:**
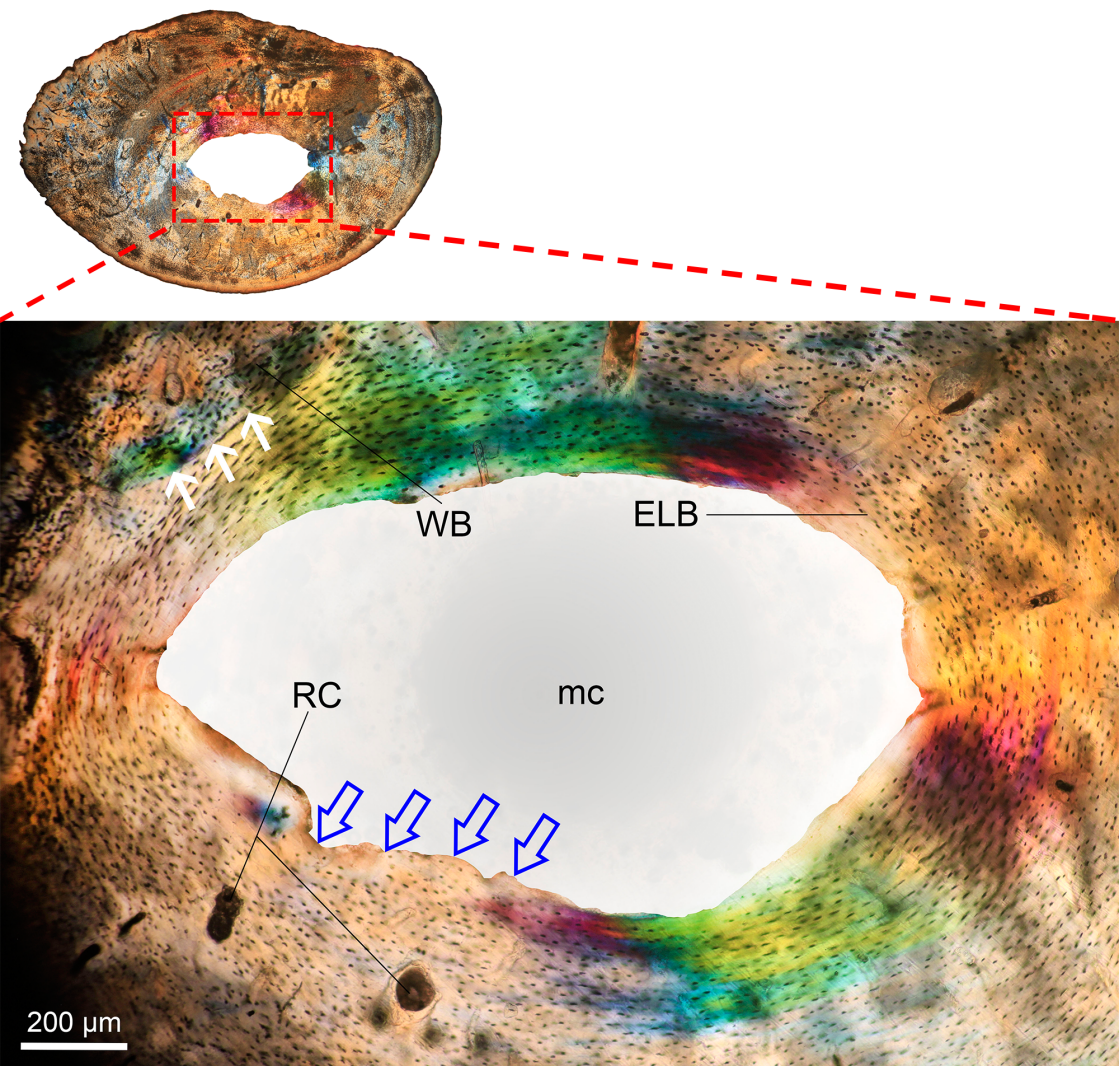
Cross section of a femur of a male of *Bathyergus suillus* (#1154) showing a relatively intact perimedullary region (photograph under polarized light). A thick layer of endosteal lamellar bone (ELB) surrounds the medullary cavity (mc). A reversal line (white arrows), endosteal surface resorption (blue outlined arrows) and small resorption cavities (RCs) are observed, but not secondary redeposition.

**Figure 10:**
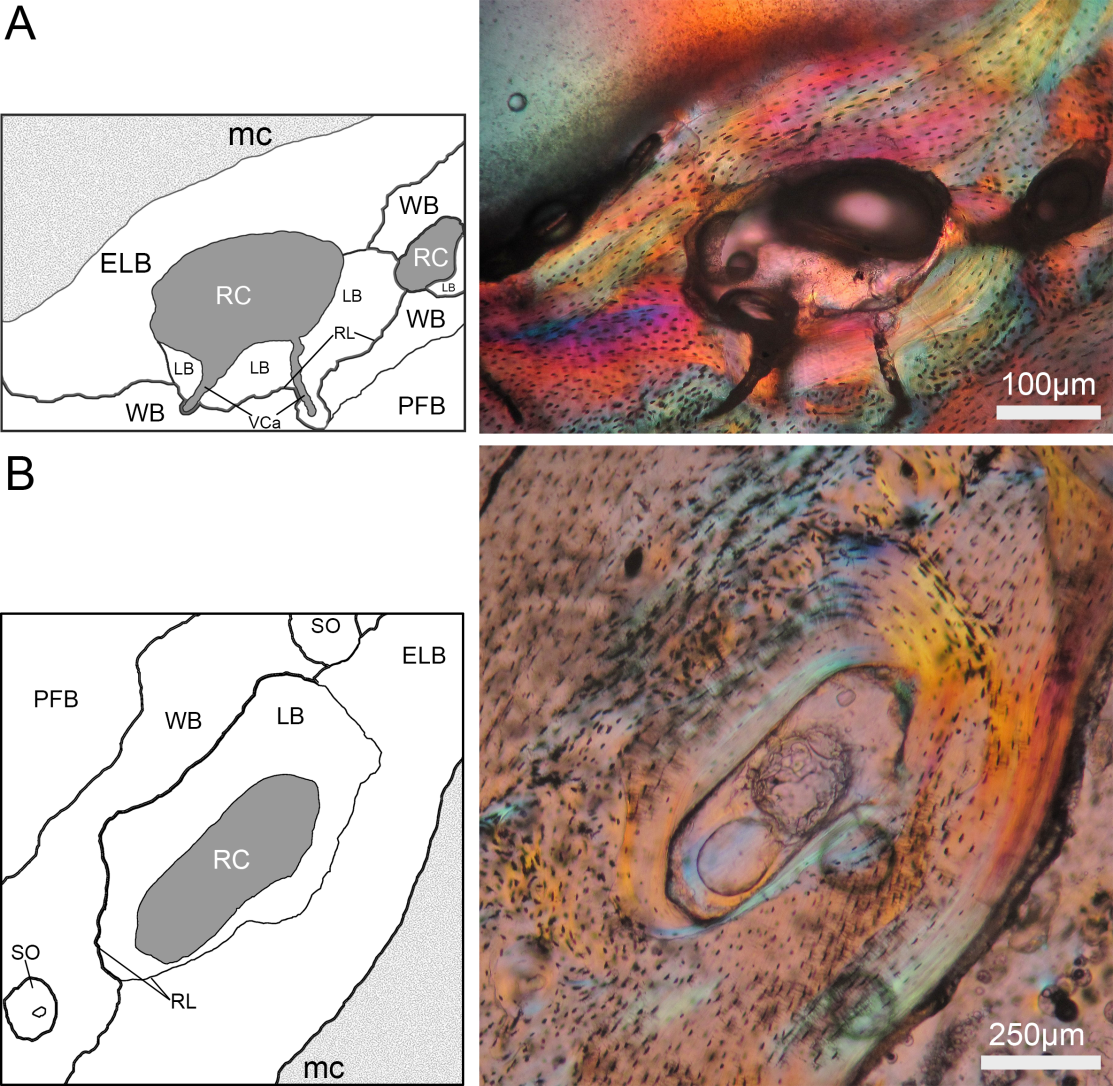
Subendosteal remodeling in females of *Bathyergus suillus* (photographs under polarized light). (A) A resorption cavity (RC) with one side partially infilled with lamellar bone (LB) (#1138); (B) RC with partial infilling of LB (#377). Abbreviations: ELB, endosteal lamellar bone; mc, medullary cavity; PFB, parallel fibered bone; RL, resorption line; SO, secondary osteon; VCa, vascular canals; and WB, woven bone.

**Figure 11:**
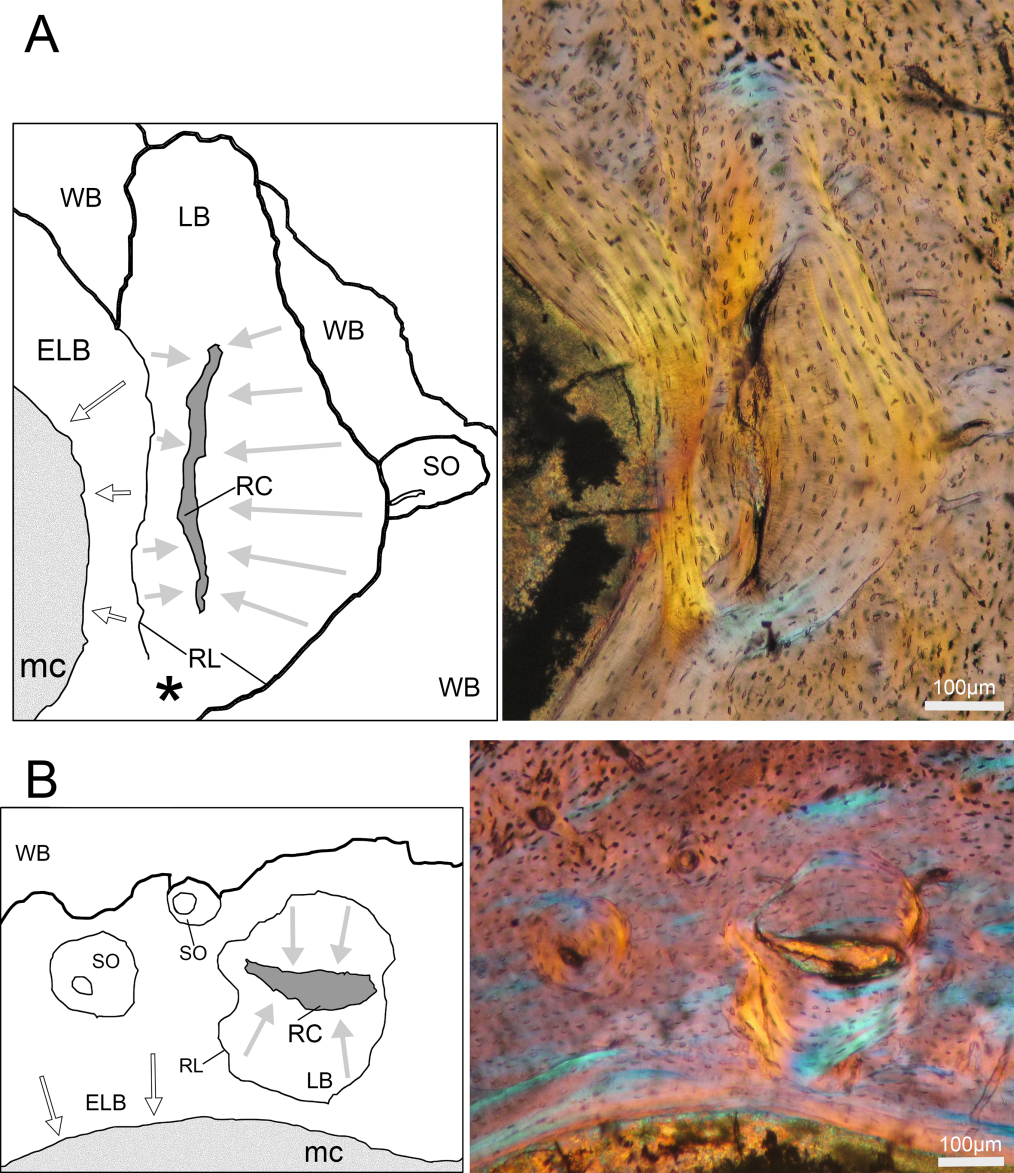
Subendosteal remodeling in females of *Bathyergus suillus* (#377; photographs under polarized light). (A) A resorption cavity (RC) with relatively complete infilling of lamellar bone (LB). The asterisk indicates a continuous deposition of lamellar bone (LB), which is also connected with the endosteally formed lamellar bone (ELB). Bone infilling is mostly deposited from the lateral sides of the RC, parallel to the endosteal margin (gray arrows); (B) RC with almost complete infilling of LB. Bone infilling is mostly deposited from upper and lower sides of the RC, also parallel to the endosteal margin (gray arrows). White arrows indicate the normal direction of the bone deposition at endosteal surfaces. See Fig. 10 for abbreviations.

Regions with secondary reconstruction (i.e., RCs showing infilling of lamellar bone) were observed in both sexes, although there are conspicuous differences between sexes. In general, the infilling of RCs can be complete, resulting in secondary osteons, or with partial infilling. The latter usually happens in enlarged RCs, which are not completely infilled with lamellar bone (Fig. 2). This last process seems to be more frequent among females only, and males did not show large RCs or extensive bone remodeling apart from secondary osteon formation. The maximum RC size of males was only about 10% of the maximum RC size of females (Table 6). For this reason, endosteal and subendosteal regions in males are mostly intact (Fig. 9). A high number of the females (54%) showed regions under active remodeling, which is evidenced by subsequent centripetal deposition of lamellar bone and the presence of reversal lines around RCs with irregular shape (Table 1; Figs. 2 and 10). Irrespective of the shape of these remodeled RCs, the centripetal infilling of lamellar bone is usually oriented relatively parallel to the margin of the medullary cavity (Fig. 11). Additionally, several reversal lines in the endosteal lamellar bone (Chinsamy-Turan, 2005) were observed. The latter indicates repeated events of bone resorption and deposition at perimedullary regions, although it is hard to say if they represent events of bone modeling (e.g., cortical drift) and/or bone remodeling. Both histomorphometric data (e.g., Es.Pm and Me.Ar; Table 3) and histological observations evidence a more pronounced endosteal remodeling in females.

## Discussion

In the present study, we used bone histology, bone microanatomy and bone histomorphometry to determine the pattern of femoral cortical bone growth and resorption of a feral population of CDMs *B. suillus*. Our observations confirm our hypotheses, that mineral content increases in *B. suillus* during postnatal aging and that they have limited intracortical and endosteal resorption, although a pattern of mineral mobilization is observed between sexes. These aspects are discussed in relation to previous reports on mammals (i.e., interspecific variation), as well as in terms of sexual differences in skeletal homeostasis (i.e., intraspecific variation).

### Cortical changes in CDMs

Femoral cortical thickening in *B. suillus* is reached mostly by periosteal apposition, as deduced from significant increments in cross sectional area, cortical area and cortical thickness during ontogeny, and most importantly, due to scarce intracortical and endosteal bone resorption (while retaining medullary cavity size) (Table 3; Fig. 3). Although this study did not assess bone growth in other regions of the femur (e.g., metaphyses or epiphyses), our results, along with previous detailed histological descriptions of mainly transverse sections, and including a few longitudinal ones (Montoya-Sanhueza & Chinsamy, 2017), suggest that the femur of CDMs quite substantially increases its bone mass, and preserves its overall bone microstructure during its entire lifespan (Figs. 3B and 3D). A similar trend has been found in naked molerats (NMs), although only individuals younger than 15 years old (50% of their lifespan) have been studied so far (Pinto et al., 2010).

The ontogenetic process of cortical bone gain in CDMs differs from the pattern of bone mass increment in humans and other mammals, which reach a plateau at young adult life (i.e., peak bone mass), and thereafter begins to decrease (Daniels et al., 1995, 1997; Jee, 2001; Lei et al., 2004; Wang, 2013). Peak bone mass in CDMs appears to occur late in ontogeny, since they reach the 50% of their cortical area (Ct.Ar) in subadult stages (age classes 4–5) (Table 3), apparently during the transition to sexual maturity (Hart et al., 2007), but continue depositing bone well into adulthood (Table 3). In humans, femoral peak bone mass is reached around the third and fourth decade, long after they have reached sexual maturity (Daniels et al., 1995, 1997; Jee, 2001; Mora & Gilsanz, 2010), while among other rodents peak bone mass occurs around three months, on the onset of puberty (Sengupta et al., 2005). Thus, like humans, CDMs continue forming bone after sexual maturity, although unlike humans, bone continues to be deposited (albeit slowly) during late stages of ontogeny. Interestingly, previous reports by Hart et al. (2007) indicate that the axial skeleton (body length) of male CDMs continue increasing during late adulthood.

Male CDMs accumulated more bone as compared to females, although females seem to show faster rates of periosteal expansion during the juvenile-subadult transition (Table 4). Since endosteal bone deposition appears to decrease in females during this stage (Figs. 3C and 3D), it is likely that this faster growth in females is mostly a result of periosteal deposition. This male-biased sexual dimorphism is similar to the pattern of bone growth observed in humans, where males reach higher cortical parameters and bone mineral density as compared to females (Silva & Jepsen, 2013; Wang, 2013). However, humans do not show sex differences in bone width and radial growth rates before puberty (Szulc, 2010). From the third decade onwards, human females show slower rates of periosteal bone expansion than males (Silva & Jepsen, 2013). This is more similar to what happens in subadult and adult CDMs (Table 3; Fig. 3B).

Continuous periosteal expansion and low bone turnover are some of the factors usually measured to establish the degree of bone quality in an individual (Grynpas, 2003), and it appears that *B. suillus* has maximized their mechanism of bone modeling to reach sustainable levels of bone quality throughout age. Interestingly, the strenuous effects that digging activities may cause are expected to be similar to those found in experimental studies with organisms subjected to dynamic loads (e.g., Martin, Burr & Sharkey, 1998; Currey, 2002). However, digging activity in CDMs does not appear to leave any considerable effect on femoral bone remodeling (e.g., increasing number of secondary osteons), since this process is kept relatively at low levels in this species. This later agrees with previous reports of other small (Enlow & Brown, 1958) and giant (Geiger et al., 2013) rodent species, which lack the formation of extensive remodeling, e.g., dense Haversian tissue. In general, these findings are relevant to understand the processes governing the acquisition and maintenance of bone during ontogeny in this and other mammals (Klein et al., 1998). However, more samples of juveniles are needed to have a better understanding of the cortical growth in early postnatal stages of development of CDMs.

### Medullary cavity expansion and bone modeling in terrestrial and subterranean mammals

This study provides strong quantitative support for the idea of positive bone modeling in CDMs, where bone formation during ontogeny considerably surpasses bone loss, almost 100-fold (Table 3; Fig. 3E). This low bone turnover is mostly a result of scarce endosteal resorption, thus differing markedly from other terrestrial mammals, which follow a more negatively unbalanced bone modeling during life, i.e., mineral loss from endosteal margins increases with age resulting in an enlarged medullary cavity size (Carrier, 1983; Sontag, 1986a, 1986b; Heinrich, Ruff & Adamczewski, 1999; Lammers & German, 2002; Morbeck, Galloway & Sumner, 2002; Chinsamy-Turan, 2005; Castanet, 2006; Young, Fernández & Fleagle, 2010; Morgan, Barnes & Einhorn, 2010; Parfitt, 2010; Silva & Jepsen, 2013; Quemeneur, de Buffrénil & Laurin, 2013; Pazzaglia et al., 2015).

The RCA (≈BC) parameter seems to be a relatively good predictor of the degree of bone thickness within femoral cross sections of small to medium-sized terrestrial mammals, since they usually have a non-trabecular microanatomy (Foote, 1916; Quemeneur, de Buffrénil & Laurin, 2013). This can be used to determine the degree of occlusion of the medullary cavity in these organisms, where low values of RCA indicate enlarged medullary cavity sizes respect to the entire cross section, while higher values would indicate a small/occluded one. In this sense, previous interspecific comparisons of BC of a wide range of cursorial terrestrial mammals have reported mean values of 0.57 ± 0.11 for the femur (*n* = 37; Supplementary data in Quemeneur, de Buffrénil & Laurin, 2013). The ontogenetic BC mean obtained for *B. suillus* (including all age classes) is 0.77 ± 0.10, with adults alone having a more constrained value, 0.83 ± 0.04 (*n* = 19) (Table 3). It should however be noted that Quemeneur, de Buffrénil & Laurin (2013) obtained BC values from different species using single somatically mature individuals of unknown sex. They also chose the thickest region of the femur, which is not necessarily the midshaft of the bone, as in this study. Thus, if the BC of these taxa would be measured in the actual midshaft of those species (i.e., 50% from distal or proximal end of the bone), it might be even lower than the reported values. Additionally, these authors included all kinds of porous spaces in the cortex, not making distinctions between void space or RCs, which suggest that the BC values may also be slightly underestimated when compared to our results. However, it is unlikely that these measures would reach the levels of compactness found in *B. suillus*, since most of their bone profile is a reflection of enlarged medullary cavities and thin cortical walls (Quemeneur, de Buffrénil & Laurin, 2013), rather than intracortical porosity.

It is expected that the quantification of the cortico diaphyseal traits in other long bones of CDMs would show a similar pattern that of the femur, especially distal elements (ulna, radius and tibia-fibula), since they show considerable reduction of the medullary cavity size (Montoya-Sanhueza & Chinsamy, 2017). This reflects the distinctive bone compactness of CDMs, and likely other bathyergids and fossorial species as well (Magwene, 1993; Straehl et al., 2013; Montoya-Sanhueza, 2014).

Considering the above, the relatively early development of femoral cortical thickening in CDMs may represent a distinctive morphological adaptation of subterranean mammals, along with their robust skeletons and usually short limb bones (Casinos, Quintana & Viladiu, 1993; Hildebrand, 1995; Stein, 2000; Kley & Kearney, 2007). In this sense, it has been reported that other subterranean species (e.g., *Ctenomys talarum*) show prenatally shaped fossorial adaptations such as incipient olecranon, wide epicondyles and distally located deltoid crest (Echeverria, Becerra & Vassallo, 2014). As scratch diggers, CDMs use principally their long-clawed forelimbs to break up soil during burrow construction, and although their hindlimbs are not directly involved in such activity, they use them in a more varied manner, providing the power for locomotion, bracing themselves during digging, assisting with soil removal by kicking the soil out of the burrow, as well as for drumming on the ground for communicating with other individuals in neighboring burrows (Bennett et al., 2009). For these reasons, it is expected that the forces acting on the hindlimb bones of CDMs can be quite diverse, involving a combination of axial compression, bending and even torsion (Biknevicius, 1993; Casinos, Quintana & Viladiu, 1993). Thus, it is most probable that the increased bone compactness of the femur reflects an adaptive functional response not only to increase mechanical resistance, but also for other associated activities of life underground.

### Intracortical resorption and sexual dimorphism

Studies of intracortical porosity in non-human mammals are still scarce and previous reports have shown a wide variability of this trait, e.g., bone loss in humans vary depending on bone site, gender, ethnicity, methodologies performed and also on the concept used to measure intracortical resorption (Stein et al., 1999; Bousson et al., 2001; Thomas, Feik & Clement, 2005; Zebaze et al., 2010). It seems that age-related bone loss (i.e., osteopenia) occurs in many mammalian species (Sumner, Morbeck & Lobick, 1989; Champ et al., 1996; Cerroni et al., 2000; Morbeck, Galloway & Sumner, 2002). Some authors have stated that all mammals undergo osteopenia during their lives, including decline in cortical thickness and area (Jee, 2001; Grynpas, 2003; Pazzaglia et al., 2015). Our results on the femur of CDMs do not support this idea, since at least in the midshaft there is no decline in cortical area with age. This most likely also applies to the rest of the limb bones of *B. suillus* (see Montoya-Sanhueza & Chinsamy, 2017).

Nevertheless, bone resorption was quantified and occurs at low levels. The greatest relative mean bone loss (intracortical porosity, Ct.Po) for males is observed in subadult stages, while for females in juvenile stages (Table 3). In general, adult stages lose about 0.85% of their cortical bone, and females can undergo around six-fold more bone loss than males (Table 3). Intracortical porosity could slightly increase if void space is included in the quantification, but these values would still be considerably lower than those reported for other mammals, e.g., healthy young humans, have porosity levels of 2–3% (Turner & Burr, 2001), and this can increase up to 18% in senile stages (Martin, Pickett & Zinaich, 1980; Feik, Thomas & Clement, 1997; Turner & Burr, 2001). Intracortical resorption has also been reported in laboratory rodents (Ferguson et al., 2003; Jilka, 2013), reaching 10% in mice (Courtland et al., 2013). All these values are accentuated with age because concurrent decline in cortical parameters (e.g., cortical thickness, cortical area) (Jilka, 2013). As mentioned before, CDMs keep forming bone during later stages of growth and do not show any sign of cortical decline, so the bone loss reflected by Ct.Po is not detrimental to its bone structure. Unfortunately, the level of intracortical porosity of rodents from feral populations is unknown, suggesting that comparisons with laboratory specimens should be treated with caution, since it is expected that porosity may increase under captive conditions due to several factors such as a lower quality diet or insufficient locomotor activity (Biewener & Bertram, 1993; Hall, 2005; Landete-Castillejos et al., 2012).

Subadults showed considerable variation in the number of RCs (n.Rc) and total resorbed area (Tt.Rc.Ar) (Fig. 10), which may indicate that high bone turnover in these stages is likely associated with gonadal hormonal fluctuations. This may be in agreement with the autoecological observations of Hart et al. (2007), which have suggested that subadults of CDMs are in a transitional stage where they may or may not be reproductive.

There is no clear ontogenetic pattern of intracortical bone loss in *B. suillus*, although sexual dimorphism was apparent. Females have higher Ct.Po (Figs. 8–9) and more secondary reconstruction than males (Table 1), although they do not show significantly higher number of n.Rc and total resorbed bone (Tt.Rc.Ar) (Table 3). Despite Tt.Rc.Ar not being significantly different between sexes, females show a tendency to increase it from subadult to adult stages, contrary to what males do (Fig. 4B). Interestingly, the variability of Ct.Po was higher in adult stages in females, and in subadult males. This may suggest differential physiological processes. Porosity decreases considerably in males with age (Table 3), suggesting that bone formation does not decrease or that bone resorption does not increase significantly during ontogeny. Similar patterns have been found in males of other rodents (Li & Klein, 1990). Females also have less n.Rc than males, which can be explained by the coalescence of RCs during extended intracortical resorption (Keshawarz & Recker, 1984). In this sense, higher degrees of n.Rc (pore density) are not synonymous with more resorbed bone. A significant interaction between the two factors analyzed in this study might be explaining this trend as well (Table 3). Thomas, Feik & Clement (2006) reported that the main contributor to intracortical porosity in human femoral cortical bone is the increase in RC area (Rc.Ar) rather than pore density. In other mammals, including other small rodents (Sissons, Kelman & Marotti, 1984; Yingling & Taylor, 2008) and humans (Goldman et al., 2009), extensive localized erosion and removal of endocortical bone may cause trabecularization of these regions, resulting in the formation of a noticeable transitional zone between the medullary cavity and the cortex (Keshawarz & Recker, 1984). This transitional (trabecular-like) zone was not particularly observed in CDMs, although the parameter S showed statistically significant differences between sexes (Table 3). This is most likely a reflection of the larger Rc.Ar in females which are extended circumferentially around the medullary cavity (Tables 5–6; Figs. 2, 5A and 8). Moreover, all the reproductive females (*n* = 12) analyzed in this study showed secondary reconstruction in subendosteal margins, except for two individuals (#1144 and # 717), which instead showed large RCs only (Table 1).

Recent and more complete studies assessing whole transversal cross sections have shown that the distribution of porosity in humans is not uniform around the midshaft and that there is a gradient in bone loss from periosteal to endosteal regions (Feik, Thomas & Clement, 1997; Thomas, Feik & Clement, 2005). Our observations in CDMs follow a similar pattern, and RCs distribute mostly in subendosteal regions, especially in regions with a high degree of vascularization (e.g., in woven and fibrolamellar bone matrices) (Fig. 5C). Nevertheless, the lateral side of the bone, which is constituted of a great proportion of fibrolamellar bone, seems not to be equally affected as woven bone. This indicates that, in general, there is a tendency of RCs to be developed more in vascularized tissues, but also relatively close to the medullary cavity region. In this sense, it has been observed that resorption usually occurs in the neutral axis of the bone where bending stress is lowest, so in case of structural alterations, these will not affect the biomechanical properties of the bone (Martin, 1991; Parfitt, 2003; Thomas, Feik & Clement, 2005).

Interestingly, juveniles did not show any signs of extensive bone remodeling, although they resorbed relatively more bone matrix than any other age class, especially the juvenile females (Table 3). Juveniles also presented a higher incidence of trabeculae than later ontogenetic stages (Table 1). It seems that the causes of bone mobilization in juveniles are different from those of adults and subadults. This may be due to the process of metaphyseal bone compaction during bone elongation, which is more accentuated at early stages of somatic growth. During this process, endosteal lamellar bone is deposited around trabeculae formed endochondrally at the metaphyses, to be subsequently relocated in to the diaphyseal region (Enlow, 1963). This results in the formation of a histologically different bone tissue, compacted coarse cancellous bone (Enlow, 1963; Chinsamy-Turan, 2005; Montoya-Sanhueza & Chinsamy, 2017). It is probable that diaphyseal midshafts in juveniles were still under this process of diaphyseal bone relocation, especially the small females, which showed thin cortices and more trabeculae. It is possible that subadult and adult stages had already completed this process, since compacted coarse cancellous bone was commonly observed in the midshaft (Table 2 in Montoya-Sanhueza & Chinsamy, 2017). In this sense, bone resorption in juveniles may reflect high bone turnover due to bone modeling (e.g., diaphyseal growth, cortical drift and/or development of the third trochanter) rather than to other adaptive processes such as bone remodeling (i.e., secondary reconstruction).

Considering that the sum of bone formation and resorption gives an estimation of the rate of bone turnover (Sontag, 1986a) (Fig. 3E), it is quite clear that the diaphyseal region of the femur has low rates of bone turnover regardless of sex. This is generally expected for cortico diaphyseal bone (Parfitt, 2002), especially compared to more distal regions with trabecular bone, where high bone turnover usually predominates (Parfitt, 2002; Dion, Fortin & Ste-Marie, 2011). Assessment of more proximal regions of the bone and inclusion of 3D visualization methodologies would expand our knowledge on this particular bone modeling in CDMs.

### Histomorphometry of RCs and sexual dimorphism

The differences in intracortical resorption described above suggest that the catabolic processes occurring between males and females differ, and to assess this we performed a quantitative histomorphometric analysis of their RCs. All the parameters analyzed showed that the RCs in females are larger and irregular in shape (non-circular), while males have smaller and more circular RCs (Table 6; Fig. 7).

Specifically, these results indicate that the RCs in males are a result of secondary osteon formation (Haversian remodeling), whilst the RCs of females appear too big to be part of the “normal” development of a secondary osteon. Actually, the maximum RC size of females was around 90% larger than the maximum RC size of males (Table 6). Females also frequently showed incompletely infilled RCs. This does not mean that females do not form secondary osteons, but rather that their development may be obscured by the considerable enlargement of RCs (Figs. 5 and 8). Although we did not quantify the density of secondary osteons, visually it appears that there are no considerable differences for these traits between sexes.

Interestingly as well, there is no formation of dense Haversian tissue in any region of the cortex in the femur of *B. suillus*, or any other long bone previously examined (Montoya-Sanhueza & Chinsamy, 2017). This may suggest that despite enduring high levels of biomechanical stresses, bone remodeling for repairing microstructural damage (Currey, 2002) is apparently not required.

The extensive resorption of bone matrices can result from different causes: (i) female reproduction (Purdie, Aaron & Selby, 1988); (ii) hibernation (Hall, 2005); (iii) malnutrition (Sissons, Kelman & Marotti, 1984); (iv) lack of mechanical load and skeletal disuse (Biewener & Bertram, 1993); as well as pathological factors (Jowsey, 1963; Rubin & Nanes, 2005). Unfortunately, it is not completely known whether these different processes have a distinct (morphological or distributional) pattern of bone loss within the cortex (Jowsey, 1963; Matheny et al., 2013). However, most of these factors could affect any of the members of a population, making it unlikely that the sex-biased histomorphometric differences observed in this study are due to these ecological (i.e., seasonality inducing hibernation, lack of resources inducing malnutrition) and biomechanical (i.e., skeletal disuse) factors. It is also unlikely that only the females were affected by a particular bone disease, since previous histological descriptions showed no anatomical indicators of it (Montoya-Sanhueza & Chinsamy, 2017).

Our findings are consistent with bone loss as a result of female mammalian reproduction, which is known to incur high mineral imbalances and skeletal deterioration during pregnancy and lactation to meet the needs of fetal development (Kovacs & Kronenberg, 1997; Kovacs, 2001, 2005; Cerroni et al., 2003). These studies reflect the relevant metabolic (reproduction-related) function of bone and its shifts in composition in females during reproduction, e.g., bone weight, bone mineral density, bone volume, ash weight, calcium content (Namgung & Tsang, 2003); quantitative microstructure (Miller et al., 1986; Tojo et al., 1998; Namgung & Tsang, 2003) and microanatomy (Ruth, 1953; Purdie, Aaron & Selby, 1988; Shahtaheri et al., 1999). Humans, rodents and carnivores have shown similar patterns of subendosteal resorption during reproduction (Purdie, Aaron & Selby, 1988; Vajda et al., 1999). In general, mineral demands related to reproduction are largely obtained from cancellous bone at epiphyses of long bones (Kovacs, 2005), although intracortical resorption at midshafts also occurs (Keshawarz & Recker, 1984; Vajda et al., 1999; Macica et al., 2016).

Thus, different lines of evidence showing that females have a higher Ct.Po, considerably larger RCs, circumferentially arranged RCs and the occurrence of secondary endosteal reconstruction, indicate differential reproductive-related skeletal homeostasis in CDMs. Along these lines, female CDMs also seems to have an improved (faster) mechanism of cortical growth prior to the start of reproduction. This latter may be related to a mechanism enabling females not to undergo skeletal deterioration during reproductive events. This is not too different from other mammals, where the formation (or preservation) of cortical bone has been observed in the midshaft of long bones in early pregnancy (Miller et al., 1986; Tojo et al., 1998). Several studies have reported how estrogen stimulates osteoblast function *in vivo* in mice, rats, rabbits and dogs (Samuels, Perry & Tobias, 1999). Thus, it is likely that the subendosteal secondary reconstruction found in females of CDMs may be regulated by estrogen, thus consisting of a mechanism for skeletal recovering after weaning to facilitate the next reproductive cycle (Ruth, 1953). This may be particularly important in a solitary subterranean species where the preservation of bone strength is important to ensure digging activity and foraging.

Although CDMs possess a different mineral metabolism as compared to most other terrestrial mammals, the reproduction-related process is quite similar (i.e., in terms of the morphology of RCs and in its location within the cortex) to that found in mammals with mineral homeostasis regulated by vitamin D. This indicates that even when the mechanism for calcium acquisition may differ among mammals, some specific shared adaptations persist. Likewise, it is probable that the degree of resorption in CDMs is not considerably high due to the highly efficient mechanism of calcium absorption, which may guarantee efficient extractions of this mineral from the diet (Buffenstein & Pitcher, 1996; Stein, 2000). A systematically controlled assessment of the reproductive process and its effects on bone structure could shed light on how some specific reproductive stages (e.g., sexual maturity attainment, pregnancy and/or lactation) affect the skeletal system of females, as compared to males.

## Conclusion

This study shows that despite having subterranean lifestyles, with limited exposure to sunlight (and hence low concentrations of vitamin D), CDMs have highly mineralized cortical walls with no evidence of bone diseases generally associated with vitamin D deficiency. It is expected that the pattern of positively imbalanced bone modeling showed by *B. suillus* is also present in other long bones (e.g., ulna, radius and tibia-fibula), as a generalized phenomenon of systemic bone thickening in this species and likely other AMs.

This study also showed that CDMs undergoes intracortical resorption and scarce remodeling, although secondary reconstruction is accentuated in females (most likely in response to reproduction). Thus, it is apparent that such intracortical resorption is unrelated to vitamin D deficiency, and that the levels of bone resorption are not detrimental to the animal. Furthermore, it appears that the resorption observed in *B. suillus* is not attributable to only one specific physiological process and that bone modeling (growth) and reproduction seems to be involved. The data presented here further indicates that although there are no negative age-related effects on mineral content, sexual dimorphism in skeletal homeostasis does play a role.

Since in general mammalian epiphyses, metaphyses and vertebrae have higher rates of bone turnover as compared to sites composed of compact bone only (Dion, Fortin & Ste-Marie, 2011), they are usually selected for histomorphometric analyses (Duque & Watanabe, 2011). However, although the diaphyseal midshaft represents the region of the femur with low rates of bone turnover, this study showed that they were responsive to the physiological effects of bone mineral metabolism. Previous studies have reported that bones with low bone turnover (e.g., diaphyseal femora and tibia) showed histomorphometric changes when compared to regions/bones with high bone remodeling, which could be related to the fact that these regions generally keep a good track record of earlier stages of bone dynamics, so any changes occurring due to catabolic processes will be detectable (Vajda et al., 1999; Dion, Fortin & Ste-Marie, 2011). Further research should be focused on examinations of more proximal/distal trabecular regions of *B. suillus*. We also suggest that future work considers 3D analysis of bone dynamics in CDMs and other extant mammals to enable a volumetric assessment of the size and extent of the development of RCs.

## Supplemental Information

10.7717/peerj.4944/supp-1 Supplemental Information 1 Histomorphometric and microanatomical measurements of cortical bone used in this study (see text).

## Additional Information and Declarations

### Data Availability

The following information was supplied regarding data availability: The samples are all housed in the Department of Biological Sciences at the University of Cape Town, South Africa.
