# Supplementary material for: Cortical bone adaptation and mineral mobilization in the subterranean mammal Bathyergus suillus (Rodentia: Bathyergidae): effects of age and sex
Source: PeerJ. 2018 Jun 11;6:e4944. doi: 10.7717/peerj.4944 (PMC6001714; doi:10.7717/peerj.4944)
Supplement: Supplemental Information 1 [file peerj-06-4944-s001.docx]

| Histomorphometric and microanatomical measurements of cortical bone used in this study (see text) | | |
| --- | --- | --- |
| Abbreviation | **Name** | **Description** |
| FL | Femoral length (mm) | Maximum distance from the proximal articular surface to the distal articular surface. |
| Cs.Wi | Cross sectional maximum width (mm) | Transverse (mediolateral) diameter of the femur at diaphysis. |
| C.Ar | Core or sectional area (mm^2^) | Total area of a cross section. |
| Ct.Ar | Cortical area (mm^2^) | Area occupied only by ossified tissue within the cross section, calculated as: C.Ar-(Me.Ar+Tt.Rc.Ar). |
| Me.Ar | Medullary cavity area (mm^2^) | Area occupied by the medullary cavity |
| Es.Pm | Endosteal perimeter (mm) | Length of the perimedullary-endosteal surface. |
| RCA | Relative cortical area | A dimensionless parameter to quantify ossified area in a cross section and is obtained from the quotient: Ct.Ar/C.Ar. |
| n.Rc | Total number of resorption cavities (per section/specimen) | Total number of resorption cavities found in a cross section. |
| Rc.Ar | Resorption cavity area (µm^2^) | Area occupied by each resorption cavity. |
| Rc.Dm | Maximum diameter of resorption cavity (µm) | Longest line joining two points of object’s outline and passing through the centroid. |
| Rc.Rn | Roundness of resorption cavity | A dimensionless measure of the roundness of each resorption cavity, calculated as: Perimeter^2^/(4 x Pi x Area). |
| Tt.Rc.Ar | Total resorbed bone area (mm^2^) | Sum of all resorption cavity areas within a cross section |
| Ct.Po | Intracortical porosity | Quotient between the fraction of resorbed bone and its cortical area, measured as: Tt.Rc.Ar/(C.Ar-Me.Ar). |
| BC | Bone compactness | A dimensionless score of bone compactness, obtained from the ratio of solid bone tissue area to total section area. (≈RCA) |
| R/t | Ratio of bone thickness | A body size-independent measure of cortical thickness, calculated as the ratio between the outside radius of the bone wall (R) and its thickness (t). |
| CDI | Cortico diaphyseal index | A body size-independent measure of cortical thickness, calculated as the thickness of the cortex divided by the radius of the cross section. |
| S | Parameter S | Represents the sigmoid curve expressed as the reciprocal of the slope at the inflexion point. |
| P | Parameter P | Distance from the center of the cross section to the transitional zone. |
